# Assessing depletion attractions between colloidal nanocrystals

**DOI:** 10.1126/sciadv.adv2216

**Published:** 2025-04-09

**Authors:** Charles K. Ofosu, Tanner A. Wilcoxson, Tsung-Lun Lee, William D. Brackett, Jinny Choi, Thomas M. Truskett, Delia J. Milliron

**Affiliations:** ^1^ Department of Chemistry, University of Texas at Austin, 2506 Speedway, Austin, TX 78712, USA.; ^2^ McKetta Department of Chemical Engineering, University of Texas at Austin, 200 E Dean Keeton St, Austin, TX 78712, USA.; ^3^ Department of Physics, University of Texas at Austin, 2515 Speedway, Austin, TX 78712, USA.

## Abstract

Adding nonadsorbing polymers to hard microsphere dispersions generates osmotic depletion attractions that can be quantitatively predicted and designed to manipulate colloidal phase behavior. Whether depletion described by classical theories is the mechanism for polymer-mediated nanosphere attractions is less evident. Colloidal hard nanospheres and nonadsorbing polymers are challenging to realize given the diverse interactions typically present in nanoparticle dispersions. Here, we use small-angle x-ray scattering to assess whether the depletion mechanism holds at the nanoscale, leveraging a recent finding that uncharged, oleate-capped indium oxide nanocrystals exhibit near–hard-sphere interactions in toluene. Classical modeling of polystyrene depletant as penetrable spheres predicts depletion-induced phase boundaries, nanocrystal second osmotic virial coefficients, and colloidal structuring in agreement with experiments for polymer radii of gyration up to 80% of the nanocrystal radius. Experimentally observed weakening of depletion interactions for larger polymer-to-nanocrystal size ratios qualitatively follows theoretical predictions that account for how polymer physics influences depletant interactions.

## Teaser

Attractions induced by dissolved polymers largely follow theoretical predictions previously tested for micrometer-scale colloids.

## INTRODUCTION

Multicomponent complex fluids feature diverse interactions that determine their assembly outcomes, from clustering and gelation to macroscopic phase formation. In solutions containing weakly interacting colloids and polymers, exclusion of polymers from the gaps between closely spaced colloids creates an osmotic pressure imbalance that manifests as an intercolloid depletion attraction. Such depletion attractions are entropically driven, reflecting the increased depletant configurations available when colloids are near contact, as described by the classical Asakura-Oosawa-Vrij (AOV) model (*1*–*4*). Polymer concentration and polymer-to-colloid size ratio, *q*, together set the strength and range of depletion interactions and have provided experimental handles for modifying the structure and properties of depletion-driven colloidal networks including inorganic nanocrystal (NC) superlattices (*5*–*8*) and colloidal gels (*9*–*14*), cellular matrices (*15*, *16*), and emulsions (*17*, *18*). Though quantitative predictions of depletion interactions for micrometer-scale colloids are available to guide design and understanding, questions about the applicability of these concepts at the nanoscale remain.

For micrometer-sized colloids, atomic force microscopy (*19*) and optical tweezers (*20*, *21*) have confirmed that distance-dependent depletion forces match those of the AOV pair potential. Thermodynamic and statistical mechanical models based on this classical picture, which assumes ideal depletants interact as penetrable spheres, accurately describe observed depletion-induced colloidal phase behavior, establishing a mechanistic foundation for structure and property prediction (*4*, *22*–*26*). Experimental validation of polymer-induced depletion attractions between nanoparticles has proven more challenging. The lack of a clear separation of length scales between nanoparticles and their surface ligands, the depletants, the depletant persistence length, and the solvent molecules violates simplifying assumptions underlying the classical models. For 10-nm nanoparticles, the short polymer chains required for depletant-to-particle size ratios in the colloidal limit (q<0.5) intrinsically have more repulsive effective interactions (*27*, *28*) than the larger polymers used with micrometer-sized colloids. Theoretical approaches that incorporate polymer physics of the depletant molecules have helped rationalize how depletion-induced nanoparticle phase behavior differs from predictions based on classical approaches (*29*–*32*). Interrogation of thermodynamic and structural consequences of nanoscale depletion interactions is also possible via light scattering and small-angle x-ray scattering (SAXS) analysis (*33*–*35*). However, isolating the contributions from depletion is problematic for most nanoparticles due to uncertainty about the influences of nonspherical shape, polydispersity, or the presence of other confounding interactions. Strong conclusions regarding the role of depletion interactions at the nanoscale require experimental investigation of shape- and size-uniform colloidal nanoparticles that behave as effective hard-sphere colloids when dispersed in a depletant-free solvent.

Recently, Ofosu *et al.* (*36*) used a combination of dynamic light scattering (DLS), SAXS, and second osmotic virial coefficient analysis to establish that sub–20-nm oleate ligand-capped indium oxide (In_2_O_3_) NCs dispersed in toluene are stabilized by repulsive effective interactions that are well approximated by a hard-sphere potential. Consistent with molecular simulations of other colloidal NC dispersions, the van der Waals attractions arising from the polarizability of the inorganic NC cores were determined to be negligible compared to favorable ligand-solvent attractions (*36*–*39*). The latter interactions also overcame weaker ligand-ligand van der Waals attractions to yield a net hard-sphere interaction between NCs. This interaction could be characterized by a single parameter, the thermodynamic NC diameter, $\sigma_{\text{HS}}$, comprising the inorganic core plus an NC size-independent contribution from the surrounding ligand shell. The effective hard-sphere interactions established by the excluded volume of the solvent-dispersed In_2_O_3_ NCs maintains colloidal stability indefinitely (*36*).

Building on these results, here we use SAXS and second osmotic virial coefficient analysis to investigate how the addition of polystyrene (PS) to In_2_O_3_ NC dispersions in toluene induces depletion-mediated changes to colloidal structuring, interactions, and phase behavior. We consider polymer concentrations up to the overlap concentration ($0≲c≲c^{*}$) and examine NC diameters and PS molecular weights that span polymer-to-colloid ratios in the range $q_{0}≲q≲1$, where $q_{0}=2/\sqrt{3}-1\approx 0.154$ is the threshold below which effects of polymers can be rigorously mapped to an effective colloidal pair potential (*40*). The experimental data for the phase boundaries and the second osmotic virial coefficients indicate that classical models validated for micrometer-sized colloids can reliably describe the effects of depletion interactions at the nanoscale for polymer-to-colloid size ratios in the colloid limit (q≲0.5) and even up to q≈0.8. In the “equal-sized” and large *q* regime, depletant polymer physics plays an increasingly consequential role, and the depletion attraction is much weaker than predicted assuming ideal, penetrable-sphere depletants. Analysis of experimental colloidal structure factors shows that the ideal depletant model underpredicts the effects of depletion attractions at lower *q* and overestimates their effects at higher *q*, trends that can be rationalized by the molecular nature of polymer depletants that we anticipate are general to polymer-mediated depletion interactions at the nanoscale. By integrating comparisons of theory and experiment across phase behavior, net colloidal interactions (assessed via the second virial coefficient), and structure, these results advance the understanding of colloidal depletion interactions at scales relevant for synthetic NCs and biomolecules including proteins and nucleic acid complexes.

## RESULTS

**Fig. 1.**
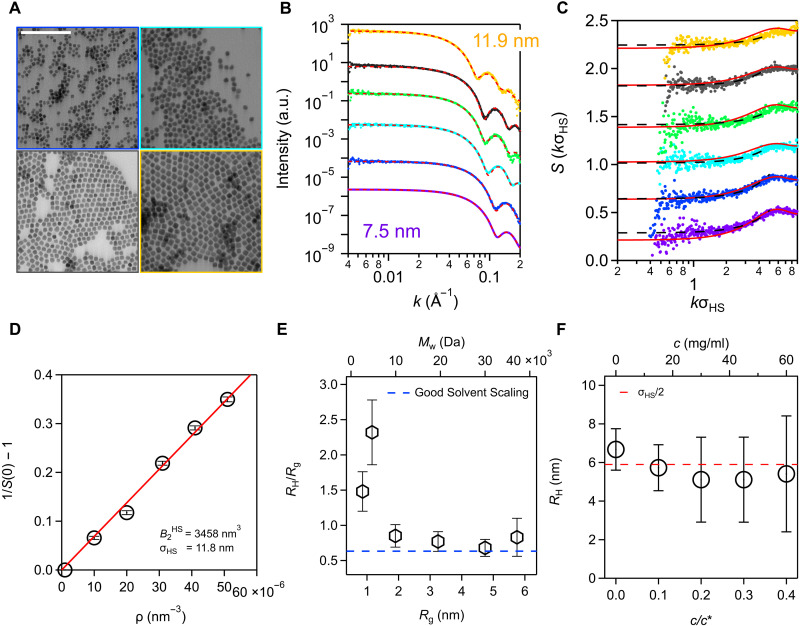
Characterization of NC-polymer mixtures. (**A**) STEM images of oleate-capped In_2_O_3_ NCs with core diameters ($\sigma_{\text{core}}$) of 7.5, 9.4, 10.4, and 11.9 nm, color coded to match the form factors in (B) (scale bar, 100 nm). (**B**) SAXS form factors P(k) of dilute NC samples in toluene (core volume fraction Φ=0.0005) with $\sigma_{\text{core}}$ from 7.5 to 11.9 nm, vertically offset for clarity. Spherical model form factor fits (dotted lines) to the data confirm the quasispherical morphology and the small size polydispersity of the NCs. (**C**) NC structure factors S(k) at a core volume fraction Φ=0.015, also vertically offset for clarity. Solid red curves represent the exact solution of the Percus-Yevick integral equation for the hard-sphere fluid at a packing fraction consistent with the experimental NC number density ρ and a hard-sphere diameter $\sigma_{\text{HS}}$ obtained from the experimental second osmotic virial coefficient ($B_{2}^{\text{HS}}$) analysis (D). Dashed curves show the quadratic fits used to extract to S(0). See fig. S2 for an NC concentration series comparison. (**D**) Reciprocal structure factor at zero wave number $S{\left(0\right)}^{-1}-1$ versus NC number density ρ. $B_{2}^{\text{HS}}$ is estimated as one-half of the slope of the linear regression, and the effective hard-sphere diameter ($\sigma_{\text{HS}}$) is determined from $B_{2}^{\text{HS}}=2{\pi \sigma }_{\text{HS}}^{3}/3$. (**E**) Ratio of polystyrene (PS) hydrodynamic radius $R_{H}$, measured using DLS, to the radius of gyration $R_{g}$, determined using the Flory-Fox equation (*78*), versus $R_{g}$. The top axis shows the corresponding molecular weight ($M_{w}$). The dashed blue line shows the good solvent scaling (*47*) for comparison to highlight the experimental deviation. (**F**) Hydrodynamic radius $R_{H}$ of oleate-capped In_2_O_3_ NCs in a 13-kDa PS solution with Φ=0.0005 and varying concentration $0≲c/c^{*}≲0.4$, with the hard-sphere radius $\sigma_{\text{HS}}/2$ obtained from $B_{2}^{\text{HS}}$ shown as the dashed line.

Oleate-capped In_2_O_3_ NCs that interact via hard-sphere–like effective pair potentials when dispersed in toluene were synthesized as described elsewhere (*36*) using a modified slow-injection method (*41*). The injection volume was controlled to produce batches of colloids with a range of diameters and narrow size distributions. Bright-field scanning transmission electron microscopy (STEM) and SAXS analysis confirmed that the NCs have quasispherical morphology and low size polydispersity (Fig. 1, A and B, and fig. S1). NC form factors P(k) measured by SAXS were well described by an analytical spherical model with core diameters ($7\;\text{nm}<\sigma_{\text{core}}<12\;\text{nm}$), accounting for a small (<9%) standard deviation in diameter for each sample. This NC size range was targeted to mitigate multiple scattering effects from the NC cores while still producing NC structure factors S(k) whose main concentration-dependent features occur at wave numbers that can be probed using our in-house SAXS instrument (*36*, *42*). For NCs of each size $\sigma_{\text{core}}$ and a series of NC core volume fractions Φ, S(k) was extracted from the NC scattering cross section using the form factor and compared to structure factors predicted by the exact solution of the Percus-Yevick integral equation for hard spheres (Fig. 1C and fig. S2) (*43*). As explained below, and consistent with earlier observations (*36*), the similarity between the experimental and predicted structure factors provides evidence supporting the approximate hard-sphere nature of the interactions between oleate-capped In_2_O_3_ NCs dispersed in toluene.

The structure factor of a hard-sphere NC dispersion depends only on NC volume fraction $\Phi_{\text{NC}}={\pi \sigma }_{\text{HS}}^{3}\rho /6={\left(\sigma_{\text{HS}}/\sigma_{\text{core}}\right)}^{3}\Phi$, which reflects the NC number density ρ and interaction diameter $\sigma_{\text{HS}}$. The latter can be recast in terms of a hard-sphere second osmotic virial coefficient, $B_{2}^{\text{HS}}$, as $\sigma_{\text{HS}}={\left(3B_{2}^{\text{HS}}/2\pi \right)}^{1/3}$. The NC radius $\sigma_{\text{HS}}$ and volume fraction $\Phi_{\text{NC}}$ reflect the combined size of the NC core and ligand shell and will be used throughout the paper to rationalize the observed phase behavior and depletant-induced interactions. For NC samples in this study (with or without added PS), we extracted the second osmotic virial coefficient $B_{2}$ from a SAXS analysis. The osmotic compressibility of an NC dispersion is related to the zero wave number structure factor ${\left(\partial \text{ln}\rho /\partial \Pi \right)}_{T}=\mathrm{\beta S}\left(0\right)/\rho$, where Π is the NC osmotic pressure, ρ is the NC number density, $\beta ={\left(k_{B}T\right)}^{-1}$, $k_{B}$ is the Boltzmann constant, and *T* is temperature (*36*, *44*). Structure factors are expected to show quadratic behavior near *k* = 0 (*45*), and thus, *S*(0) were determined by fitting a quadratic function to S(k) data at low wave number (Fig. 1C and fig. S3). Under dilute NC concentrations, a virial expansion of Π yields

$$\frac{1}{S\left(0\right)}-1=\beta {\left(\frac{\partial \Pi }{\partial \rho }\right)}_{T}-1=2B_{2}\rho +O\left(\rho^{2}\right)$$ (1)

The second equality, accurate to linear order in ρ, is used to estimate $B_{2}$ from *S*(0) by linear regression (Fig. 1D and fig. S4). The analytic Percus-Yevick hard-sphere structure factor predictions based only on measured NC number density ρ and interaction diameter $\sigma_{\text{HS}}$ approximately match experimental structure factors for NCs of different number densities and diameters with no adjustable parameters (Fig. 1C and fig. S2). Establishing this hard-sphere behavior of NC dispersions without polymer is crucial for isolating and understanding the effects of added polymer on colloidal interactions.

Linear PS depletants were studied with average molecular weights in the range $1.3\leq M_{w}\leq 35$ kDa and narrow distributions characterized by small weight average–to–number average molecular weight ratios ($M_{w}/M_{n}\leq 1.10$). These molecules adopt radii of gyration of $0.9≲R_{g}≲5.8$ nm in toluene, leading to size ratios $q=2R_{g}/\sigma_{\text{HS}}$ spanning 0.15≲q≲1 for $\sigma_{\text{HS}}=11.8$ nm, which encompasses most of the “colloid limit” (q≲0.5) for depletion interactions and crosses over into the “equal-sized” regime (0.5≲q≲2), where polymer physics plays an increasingly important role (*4*). At room temperature, toluene is a good solvent for PS (*46*). However, the hydrodynamic radius measured by DLS agrees with the expected large-polymer scaling with radius of gyration (i.e., $R_{H}/R_{g}\approx 0.63$) (*47*) only for chains of molecular weight 13 kDa and larger (Fig. 1E and Table 1). Shorter chains, necessary to access lower *q* for nanoscale colloids, have a higher value of $R_{H}/R_{g}$. Lower–molecular weight chains are also less flexible due to their relatively small number of Kuhn segments (*48*). Dynamically, they display small molecular behavior (*49*) and exhibit more repulsive effective center-of-mass interactions with each other compared to longer polymers (*27*, *28*). As discussed below, for the conditions studied here, such properties may be expected to strengthen depletion attractions and depletion-induced structuring between nanoparticles at low *q*.

**Table 1.** Linear polystyrene depletants used in this study. The radius of gyration $R_{g}$ was inferred from the intrinsic viscosity of PS in toluene ${\left[\eta \right]}^{\text{tol}}=9.27\times 10^{-3}M_{w}^{0.734}\;\text{ml}/g$ (*79*), and the hydrodynamic radius $R_{H}$ was obtained from dynamic light scattering (DLS) measurements and the Stokes-Einstein relation. PS molecular weight ($M_{w}$) and polydispersity index ($M_{w}/M_{n}$) were provided by the supplier. $N_{k}$ represents the number of Kuhn segments per polymer chain, with the Kuhn segment length for PS taken as 1.8 nm (*80*). The critical polymer overlap concentration, $c^{*}$, was calculated using the ideal volume of a polymer coil: $c^{*}=3M_{w}/\left(4\pi R_{g}^{3}N_{A}\right)=3\Phi_{0}/\left(4\pi N_{A}{\left[\eta \right]}^{\text{tol}}\right)$ where $N_{A}$ is Avogadro’s number and $\Phi_{0}$ is the Flory constant ($3.67\times 10^{24}$ ${\text{mol}}^{-1}$). Here, *q* is calculated at $\sigma_{\text{HS}}=11.8$ nm.

*M*_w_ (kDa)	*M*_w_/*M*_n_	[η]^tol^ (ml/g)	*R*_g_ (nm)	*N*_*k*_	*R*_H_ (nm)	*q*	*c*^∗^ (mg/ml)
1.3	1.10	1.8	0.9	1.7	1.3	0.15	813.0
2.2	1.06	2.6	1.2	2.9	2.7	0.20	552.6
5.15	1.04	4.9	1.9	6.9	1.6	0.32	296.0
13	1.06	9.7	3.3	17.3	2.5	0.55	150.0
25	1.06	15.7	4.7	33.3	3.2	0.80	92.8
35	1.10	20.1	5.8	46.7	4.8	0.98	72.5

For the classical depletion attraction mechanism to prevail, depletants should interact weakly with colloidal NCs, avoiding adsorption to NC-capping ligands or surface defects. The measured hydrodynamic radius $R_{H}$ of the oleate-capped In_2_O_3_ NCs showed no evidence of polymer adsorption, attaining values that were in close correspondence with the hard-sphere interaction radius $\sigma_{\text{HS}}/2$ for all polymer concentrations, establishing consistency with the second osmotic virial coefficient analysis (Fig. 1F). These results are consistent with the expectation that there are no strong attractive interactions between the PS depletants and oleate-capped NCs, which simplifies interpretation of depletion interactions in this system.

### Phase boundaries

Increasing the concentration of depletants in a colloidal dispersion strengthens the depletant-mediated colloidal attraction, ultimately driving macroscopic phase separation into a dilute and a concentrated colloidal fluid or ordered solid phase. To assess whether depletion attractions are responsible for the experimentally observed phase transitions, we first tested whether models for depletion thermodynamics can predict the boundary between single- and two-phase regions on the phase diagram. We used classical free volume theory (FVT) (*23*, *24*), which treats colloids and depletants as spheres of diameter $\sigma_{\text{HS}}$ and $q\sigma_{\text{HS}}=2R_{g}$, respectively. As in the AOV model (*1*–*4*), colloids in FVT do not overlap with other colloids or with depletants, but depletants are treated as ideal and fully interpenetrable. Using a semigrand canonical framework that accounts for the space accessible to the depletant sphere centers and unequal depletant partitioning between coexisting colloid phases, FVT has successfully predicted depletion-mediated phase behavior for polymers and larger colloids with q≲0.5 (*4*, *26*). To treat colloids and polymeric depletants with higher *q*, FVT must be generalized (i.e., GFVT) to incorporate effects of polymer concentration and polymer-solvent interactions on the osmotic pressure and the thickness of the zone around each colloid that is free of depletant centers (*26*, *50*–*52*).

**Fig. 2.**
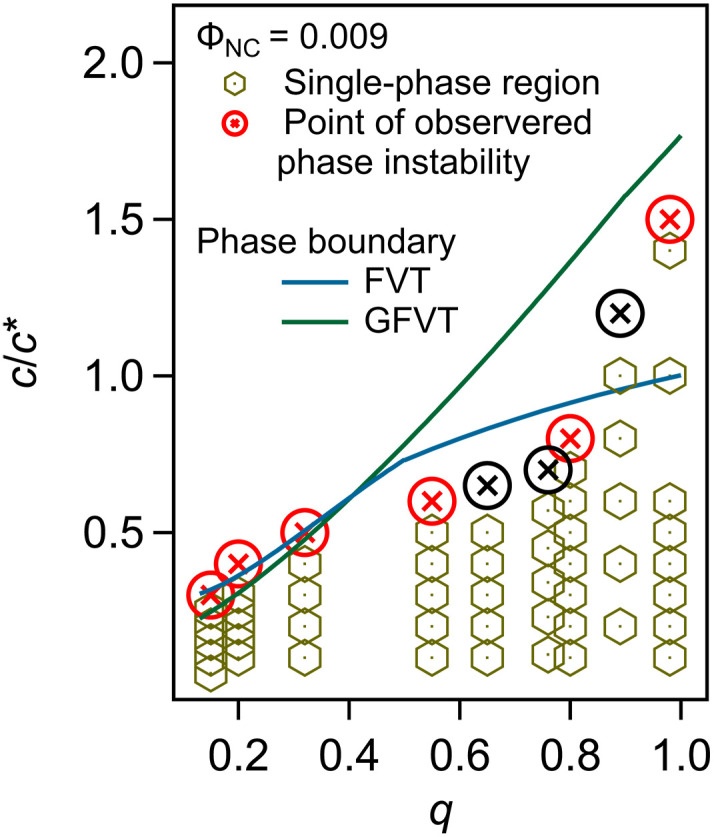
Comparison of experimental and theoretical phase boundaries. Symbols indicate experimental conditions in terms of reduced PS concentration $c/c^{*}$ and PS-to-NC size ratio $q=2R_{g}/\sigma_{\text{HS}}$. Hexagons and circles denote observed single-phase and phase-separated state points, respectively. Red symbols indicate phase boundaries with constant $\sigma_{\text{HS}}=11.8$ nm and different depletant $M_{w}$ (Table 1), while black symbols are phase instability points with fixed polymer $M_{w}=25$ kDa and $\sigma_{\text{HS}}$ from 10.6 to 14.4 nm. Curves show predictions of phase boundaries by FVT and GFVT, denoting how *q* affects the highest attainable $c/c^{*}$ in the single-phase dispersion.

Experimentally measured depletion-induced phase separation boundaries of oleate-capped NCs in toluene were determined by visual inspection as the PS concentration *c* was gradually increased. The concentrations $c/c^{*}$ (i.e., polymer volume fraction) required to cause cloudiness indicative of NC aggregation were observed, while holding $\Phi_{\text{NC}}$ fixed for various size ratios in the range 0.15≲q≲1 (Fig. 2). The size ratios were set by either choosing a single NC diameter $\sigma_{\text{HS}}$ and exploring polymers with different $M_{w}$ (and hence varying $R_{g}$) or by fixing the polymer $M_{w}$ and studying NCs with different $\sigma_{\text{HS}}$. The experimental phase boundary shows that the single-phase fluid is stabilized relative to the two-phase region with increasing *q* (i.e., higher $c/c^{*}$ is required at higher *q* to induce phase separation), whether due to increasing polymer molecular weight or decreasing NC diameter. This trend indicates that depletion interactions are weakened with increasing *q*, which is in excellent agreement with earlier experimental results, theoretical predictions, and simulated phase boundaries when studying depletion in polymer-colloid mixtures with the same *q* range, but considering colloids with diameters 10 to 100 or more times larger than the NCs studied here (*14*, *30*).

The classical FVT approach (fig. S5) predicts the phase boundary quantitatively in the colloid limit (q≲0.5). At higher *q*, for polymer depletants approaching the size of the colloids (q≈1), the phase boundary lies at higher polymer concentration, crossing into the semidilute ($c>c^{*}$) concentration range, as predicted by GFVT. The experiments show that the crossover of the phase boundary from dilute to semidilute is sharper than is anticipated by the models, occurring rather abruptly in the range 0.8≲q≲1, with FVT providing a qualitatively accurate prediction of the phase boundary for polymer-to-NC size ratios up to this limit. Below, we discuss how SAXS provides insights into the microscopic depletion-mediated interactions and structuring that underlie these trends.

### Second osmotic virial coefficients

**Fig. 3.**
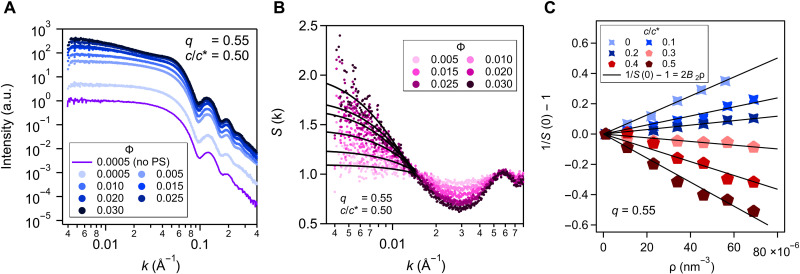
SAXS-based $B_{2}$ analysis for polymer-mediated NC attractions. (**A**) SAXS scattering profiles of oleate-capped In_2_O_3_ NCs at various Φ and constant 13-kDa PS concentration ($c/c^{*}=0.50$). The purple curve (offset for clarity) shows the sample without added PS. (**B**) NC structure factors S(k) for the intensities in (A). Black curves are Lorentzian fits in the low-*k* region to estimate S(0). (**C**) $S{\left(0\right)}^{-1}-1$ versus NC number density ρ for varying $c/c^{*}$ at q=0.55. Blue symbols ($0\leq c/c^{*}\leq 0.2$) represent repulsive NC interactions, while red data points ($0.3\leq c/c^{*}\leq 0.5$) indicate attractive NC interactions. Black lines are linear fits, with slope $2B_{2}$.

Adding polymer depletant to an NC dispersion modifies the effective colloid-colloid interaction, *U*(*r*), and the equilibrium thermodynamics of the dispersion. The second osmotic virial coefficient, which quantifies the integrated effects of depletion interactions for the osmotic pressure, $B_{2}=\left(1/2\right)\int_{0}^{\infty }r^{2}\left\{1-\text{exp}\left[-\mathrm{\beta U}\left(r\right)\right]\right\}dr$, can be characterized experimentally by SAXS analysis via Eq. 1 (*30*, *31*, *33*). Here, we extract $B_{2}$ from measured scattering cross sections of oleate-capped NCs for a series of core volume fractions 0.005≲Φ≲0.030 with fixed $c/c^{*}$ (Fig. 3). To isolate the contribution from depletion interactions, we consider the reduced second virial coefficient, $B_{2}^{*}=B_{2}/B_{2}^{\text{HS}}$, normalized by $B_{2}^{\text{HS}}$ obtained from measurements of NC dispersions without added polymer (*53*).

In the dilute NC limit, increasing Φ at constant $c/c^{*}$ enhances structural features promoted by the polymer-mediated colloid-colloid interactions. For example, at $c/c^{*}=0.50$ and a size ratio of *q* = 0.55, increasing NC concentration leads to more intense scattering at k→0, a higher value of *S*(*0*) (i.e., osmotic compressibility), and a negative $B_{2}$ (Fig. 3). To quantify how $B_{2}$ depends on polymer-to-NC size ratio and polymer concentration, scattering cross sections, structure factors *S*(*k*), and $B_{2}^{*}$ values were analyzed for at least five $c/c^{*}$ values for each *q*. For the S(k) with rising values as k→0, the quantity S(0) was estimated by fitting the structure factors to Lorentzian functions (Fig. 3B and figs. S6 to S10) (*54*)

$$S\left(k\right)\approx \frac{S\left(0\right)}{1+{\left(\mathrm{k\xi}\right)}^{2}}$$ (2)

The change in the slope of $S{\left(0\right)}^{-1}-1$ with NC number density ρ from positive to negative with increased depletant concentration (Fig. 3C) signals the expected change in the net colloidal pair interaction from repulsive (positive $B_{2}^{*}$) to attractive (negative $B_{2}^{*}$).

To assess how these measurements align with expectations based on theory, we compared measured $B_{2}^{*}$ to that computed from *U*(*r*) of the AOV model (*1*, *3*, *55*). We also compared the experimental results for $B_{2}^{*}$ with those determined from virial expansions of osmotic pressure in $\Phi_{\text{NC}}$ using FVT or GFVT (figs. S11 to S13) (*56*, *57*). Of these, FVT is the simplest, but it is expected to be least accurate for $B_{2}^{*}$. It predicts a linear dependence of $B_{2}^{*}$ on $c/c^{*}$ (*56*, *57*), which is valid at low $c/c^{*}$ but loses accuracy at intermediate and higher polymer concentrations where depletion attractions become stronger. GFVT improves the classical FVT by including consequences of polymer interactions that become important at high polymer concentrations, but it is not expected to improve the accuracy for intermediate $c/c^{*}$ below the overlap concentration (i.e., for most of the parameter space explored in this study). The AOV model has the same physical basis as FVT (hard colloids and ideal, interpenetrable depletants), but it is more suitable for predicting $B_{2}^{*}$ because it is specifically formulated in terms of the effective colloid-colloid pair potential, *U*(*r*). The $B_{2}^{*}$ prediction from AOV, while expected to be more accurate than FVT and GFVT at intermediate depletant concentrations, must lose predictive power when the polymeric nature of the depletant becomes important, e.g., when the depletants becomes comparable in size to the NCs or at polymer concentrations approaching the overlap concentration.

**Fig. 4.**
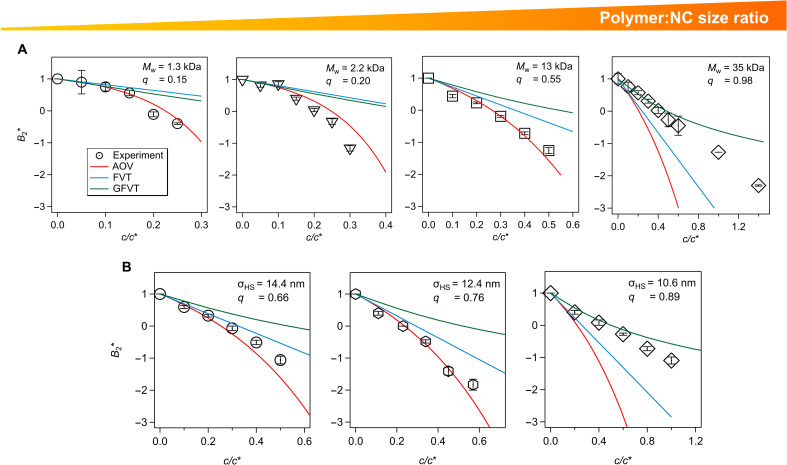
Comparison of experimental $B_{2}^{*}$ to theoretical predictions. $B_{2}^{*}=B_{2}/B_{2}^{\text{HS}}$ versus $c/c^{*}$ for 0.15≤q≤0.98 where *q* increases via (**A**) increasing $R_{g}$ at a constant $\sigma_{\text{HS}}=11.8\;\text{nm}$ and (**B**) decreasing $\sigma_{\text{HS}}$ at a constant $R_{g}=4.7\;\text{nm}$, corresponding to $M_{w}=25\;\text{kDa}$. Predictions based on the AOV colloid-colloid potential (AOV) and virial expansions of osmotic pressure in $\Phi_{\text{NC}}$ of FVT and GFVT are also shown.

The predictions of the AOV model indeed show good agreement with the experimental $B_{2}^{*}$ results across a wide range of size ratios 0.15≲q≲0.8, regardless of whether *q* is modified by varying depletant $R_{g}$ at constant NC size (Fig. 4A) or by varying $\sigma_{\text{HS}}$ for a fixed depletant $M_{w}$ (Fig. 4B). These AOV predictions for $B_{2}^{*}$, especially when viewed together with the successful FVT predictions for the phase boundary in Fig. 2, offer evidence that classical depletion theories can provide an adequate description of polymer-mediated interactions at the nanoscale, even with their simplistic treatment of depletants as noninteracting. For q≳0.8, consistent with the phase behavior shown in Fig. 2, the importance of polymer interactions in determining $B_{2}^{*}$ becomes evident. Strong deviations from classical predictions are observed as the depletion attractions substantially weaken, and the measured $B_{2}^{*}$ values lie well above AOV and FVT predictions (Fig. 4). These $B_{2}^{*}$ deviations are qualitatively captured by GFVT, as they were for the phase behavior, although quantitative deviations between GFVT and experiment persist for 0.8≲q≲1. As alluded to above, simplifications of both FVT and GFVT prevent them from accounting for the effects of multipolymer interactions on $B_{2}^{*}$, which are important for capturing experimental trends at intermediate $c/c^{*}$, despite their ability to provide an approximate description of the overall phase behavior.

One minor deviation from experiments apparent in the AOV predictions for $B_{2}^{*}$ occurs for the lower $M_{w}$ PS depletants, where AOV predicts less attractive colloidal interactions with increasing polymer concentration. This trend is apparent across multiple *q* values for NC-PS mixtures with various NC sizes containing the 2.2-kDa PS as the depletant (Fig. 4A and fig. S14). The stronger attractions in NC-PS systems with lower $M_{w}$ compared to classical depletion predictions suggest that these polymers act as more effective depletants than ideal, penetrable spheres. This behavior may arise because low-$M_{w}$ polymers, which comprise a small number of Kuhn lengths, act like semiflexible chains (*48*) and have stronger effective center-of-mass repulsions than longer polymers (*27*, *28*). There is evidence from simulation and theory that reduced flexibility in polymer depletants (i.e., fewer Kuhn lengths per chain) can produce stronger depletion attractions compared to flexible chains with the same $R_{g}$ (*58*, *59*). Density functional theory (*60*) and integral equation theory calculations (figs. S15 to S19) also show that increasing the depletant-depletant exclusion diameter can give rise to stronger polymer-mediated colloidal attractions.

### Colloidal structuring from SAXS

**Fig. 5.**
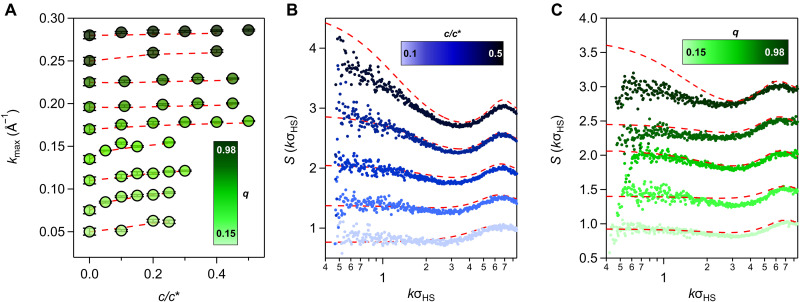
Depletion-induced colloidal structuring: Experiments and AOV model predictions. (**A**) Primary peak positions $k_{\text{max}}\left(A^{-1}\right)$ from AOV model (red dashed) and from split-Gaussian fits of experimental *S*(*k*) primary peak (symbols) versus $c/c^{*}$ with symbol color indicating *q*. All data are for $\Phi_{\text{NC}}=0.05$, and $10\leq \sigma_{\text{HS}}\leq 14.5\;\text{nm}$ with varying $M_{w}$ of PS. Error bars represent 95% confidence interval of the standard fitting errors. (**B**) Structure factors from the AOV model (red dashed) and from experiments (symbols) at $\Phi_{\text{NC}}=0.05$, $\sigma_{\text{HS}}=11.8\;\text{nm}$, *q* = 0.55, and $0\leq c/c^{*}\leq 0.50$ from light to dark blue. (**C**) Structure factors from the AOV model (red dashed) and from experiments (symbols) at $\Phi_{\text{NC}}=0.05$, $\sigma_{\text{HS}}=11.8\;\text{to}\;12.4\;\text{nm}$, varying *q,* and *c*/c^*^ selected to maintain a constant experimental $B_{2}^{*}≃0.01$ to highlight the *q*-dependent deviations of experiments from the AOV model. In all panels, the bottommost data and model correspond to the vertical axis values, while the results above are vertically offset for clarity.

Beyond implications for macroscopic phase behavior and osmotic compressibility, depletion interactions also affect the organization of the colloids across length scales, even for polymer concentrations low enough to avoid aggregation and phase separation. The oleate-capped In_2_O_3_ NCs investigated here are uncharged (fig. S20). Their neutrality is advantageous in that it avoids the complication of additional, long-range repulsions arising from electrostatics. Combining short-range depletion attractions and long-range repulsions can promote the formation of self-limited clusters (*61*), which are interesting but would complicate the interpretation of *S*(*k*) (*62*–*66*). Clustering can also occur due to depletion interactions at higher NC concentrations near percolation or gelation transitions (*9*, *10*, *14*), but such conditions were intentionally avoided in this study to focus on understanding the most fundamental structural implications of depletion interactions. Accordingly, there is no evidence of intermediate-range order due to clustering in the structure factors of the single-phase fluid state points reported here (Figs. 1C, 3B, and 5, B and C and figs. S2, S3, S6 to S10, and S21 to S30). Instead, our analysis examines how depletion attractions influence the position of the structure factor’s primary peak $k_{\text{max}}$ by varying reduced polymer concentration $c/c^{*}$ for various fixed polymer-to-NC size ratios *q*. How *q* modifies the shape of *S*(*k*) at a constant attraction strength is also explored.

The addition of depletant leads to a common structural evolution as $c/c^{*}$ increases, evident in *S*(*k*) for all NC dispersions studied. A primary peak, located at $k_{\text{max}}\approx 2\pi /\sigma_{\text{HS}}$, corresponding to interparticle separations of $2\pi /k_{\text{max}}\approx \sigma_{\text{HS}}$ (first-coordination shell or “cage” particles), becomes more pronounced, accompanied by a deepening correlation well at lower *k* and a rise in *S*(*k*) as k→0. For all *q* values, $k_{\text{max}}$ increases modestly with $c/c^{*}$ (Fig. 5A), suggesting that the short-range, locally ordered environment around the NCs draws closer in response to increasing depletion attractions (*67*). AOV predictions based on the reduced polymer concentration, $c/c^{*}$, match the experimental $k_{\text{max}}$ values within a 95% confidence interval, even for high *q* where experimental $B_{2}^{*}$ values deviate strongly from the AOV model (Fig. 5 and figs. S21 to S30). This close match between experimental and AOV $k_{\text{max}}$ suggests that the characteristic separation distance derived from the primary peak of *S*(*k*) is less sensitive than *S*(*0*) (or $B_{2}$) to the polymeric physics of the depletant and its impact on depletant-depletant and depletant-NC interactions.

Comparison of the shape of the experimental S(k) to that of the AOV model reveals deviations from the structure predicted by AOV not apparent in the analysis of $k_{\text{max}}$ dependencies on $c/c^{*}$ and *q*. For intermediate *q* = 0.55, at the crossover between the colloidal limit and the equal-sized regime, we observe excellent agreement between experiments and AOV predictions across $c/c^{*}$ without adjustable parameters (Fig. 5B), capturing both the primary correlation peak and the low *k* behavior. Comparing experimental and AOV S(k) across different *q* and $c/c^{*}$ selected to maintain the same experimental pairwise attraction strength ($B_{2}^{*}≃0.01$), however, reveals deviations (Fig. 5C), just as we saw deviations in $B_{2}^{*}$ trends with $c/c^{*}$ for *q* values near the upper and lower ends of the range we studied (Fig. 4). On the basis of the theory-experiment *S*(*k*) comparisons, the AOV model substantially overestimates the attractions present at q≈1, where the specifics of the real interactions among polymers and between polymers and particles become important. The AOV model also mildly underpredicts the attractions at low *q*. As discussed above, this deviation may be because the low-*q* limit for nanoscale colloids can only be realized with short chains. Short-polymer depletants, because of their reduced flexibility and stronger interchain effective repulsions, may be more effective depletants than ideal, interpenetrable spheres with the same $R_{g}$.

## DISCUSSION

Depletion interactions between micrometer-scale colloids have been extensively characterized by a combination of techniques including optical tweezers and microscopy, atomic force microscopy, and light scattering. The length scales present in such systems—the solvent, the polymer depletants, and the colloids—are well separated from each other. This separation leads to simplifying assumptions foundational to classical depletion interaction theories, whose predictions have been extensively validated by experiment. Though there are open questions on how other types of interactions between micrometer-scale colloids combine with or modify depletion interactions (*4*), the latter have become an integral part of the self-assembly toolkit. Making similar strides for understanding and leveraging depletion interactions to direct assembly of nanoscale colloids or biological molecules like proteins, where the separation of scales is less evident, would represent a fundamental advance with considerable practical implications. Although there is already ample evidence of depletion-like interactions between colloids with diameters of 10 nm and smaller, tests that enable the kind of mechanistic understanding needed for interpretation and design have been more challenging to advance due to limitations of nanoscale characterization tools and a lack of model nanosized colloids with uniform size and shape and neutral (e.g., hard-sphere–like) interactions. Here, we have taken advantage of a recent discovery that uncharged, oleate-capped In_2_O_3_ NCs with low polydispersity exhibit near–hard-sphere–like interactions when dispersed in toluene (*36*), allowing them to serve as a model colloid for testing the applicability of classical depletion theories at the nanoscale.

We quantitatively characterized depletion-mediated colloidal structure and interactions of the NCs with PS depletants in toluene using SAXS and DLS. To overcome the indirect nature of SAXS (compared to optical microscopy used for larger colloids), we combined thermodynamic analysis ($B_{2}^{*}$) and structural analysis [*S*(*k*)], and investigated trends across various polymer molecular weights and NC sizes. We considered a range of polymer-to-NC size ratios (0.15≲q≲1) common to studies of nanoparticle dispersions, spanning from the conventional colloid limit to the equal-sized regime. By analyzing interactions in the single-phase regime, leading up to phase separation, this type of analysis goes beyond, and augments, the conventional focus on indirect tests of depletion interactions via phase boundary analysis, allowing us to identify nanoscale-specific limitations and insights. We show that the classical AOV picture with interpenetrable sphere depletants is largely adequate for describing the phase boundaries (predicted by FVT) as well as the second osmotic virial coefficients and the structure factors in the fluid phase for 0.15≲q≲0.8. This agreement with AOV for the conditions studied is consistent with previous studies of micrometer-sized particles, where deviations from the classical AOV picture are observed at higher polymer concentrations, larger polymer-to-colloid size ratios, or high colloid volume fractions (*20*, *26*, *30*, *31*). The one caveat is that the depletion interactions for the lower end of the *q* range studied are stronger at higher polymer concentrations than expected based on AOV, possibly due to the limited flexibility and nonideal depletant-depletant interactions characteristic of short polymers. These results suggest that polymer architecture, including flexibility, may serve as an effective parameter to more finely control the strength of depletion interactions, especially at the nanoscale.

For larger polymer-to-NC ratios 0.8≲q≲1, we find phase boundaries, second osmotic virial coefficients, and structure factors consistent with a crossover to much weaker depletion interactions. This behavior qualitatively mirrors what is anticipated by GFVT due to the increased importance of the polymer-polymer and polymer-nanoparticle interactions when the depletant size approaches that of the colloid. The transition to weaker interactions is sharper than is anticipated by GFVT and highlights the consequences of selecting different depletant sizes in a regime that is commonly encountered in nanoparticle-polymer mixtures (*16*, *32*, *33*, *68*). Our results also highlight that substantial errors can be made if AOV is used to describe nanoscale depletion interactions for higher size ratios outside its range of applicability.

In detailing how depletion interactions manifest in model nanoscale colloids, the results of our study provide a crucial reference system and framework for understanding and interpreting more complex nanoparticles, like proteins (*29*, *35*, *69*, *70*). This approach will also be useful for quantitatively characterizing the effects of surface modifications, e.g., block copolymer wrapping (*32*, *71*), which can endow particles with controlled charge or effective polymer graft densities and molecular weights that modify polymer-mediated interactions (*32*). The applicability of classical depletion theories to describe depletion by linear polymers at the nanoscale also raises the possibility of tuning depletant chain flexibility or even topology to control nanoparticle interactions and phase behavior (*72*).

## MATERIALS AND METHODS

### Synthesis and characterization of NCs

In_2_O_3_ NCs were synthesized on a Schlenk line by modifying a slow growth procedure (*41*). A precursor solution containing In(III) acetate in 10 ml of oleic acid was degassed under vacuum for 15 min at 110°C, followed by a 5-min N2 purge. This cycle was done a total of three times, and heated under N2 to 150°C for 2 hours. The solution was then slowly injected into 13 ml of oleyl alcohol at 290°C under N2. NC size was varied by changing precursor injection volume. After synthesis, NCs were washed five times with ethanol and dispersed in hexane. STEM and SAXS were used to confirm the morphology and determine the size and size distribution. Hydrodynamic radii of the NCs were determined from DLS and the calculated polymer concentration–dependent solution viscosity (*73*).

### FVT/GFVT calculations of phase boundaries and second osmotic virial coefficients

In FVT, a colloid-depletant dispersion is in contact with a depletant reservoir, separated by a membrane that is permeable to only depletant and solvent (*24*). Colloids are modeled as hard spheres, while depletants are represented as fully interpenetrable spheres with no interdepletant interactions. Solvent interactions are only implicitly included in the other model parameters (i.e., colloid or depletant diameters). The semigrand potential of the colloid-polymer system is computed by considering the osmotic equilibrium between the polymer in the colloid-polymer dispersion and the reservoir. The GFVT extends FVT by incorporating polymer properties into the depletant-depletant and colloid-depletant interactions. Using the equations of state from FVT and GFVT, phase boundaries and second osmotic virial coefficients can be calculated through standard thermodynamic relations. More details can be found in the Supplementary Materials and in (*4*, *26*).

### AOV model structure factor calculations

AOV model structure factors were computed for NCs interacting via the AOV potential by numerically solving the Ornstein-Zernike equation with approximate closures via pyPRISM (*74*). We similarly calculated structure factors for two-component NC-depletant systems with penetrable hard-sphere depletants to check the accuracy of using the effective AOV potential. Last, we used Brownian dynamics simulations using the Highly Optimized Object-oriented Many-particle Dynamics-Blue Edition (HOOMD-Blue) simulation toolkit (*75*, *76*) to model NCs interacting via the AOV potential and the Heyes-Melrose hard-sphere interaction potential (*77*) to validate the accuracy of the Ornstein-Zernike solutions to the AOV model. More details about the AOV model structure factor calculations and validations can be found in the Supplementary Materials.
