# Supplementary material for: Assessing depletion attractions between colloidal nanocrystals
Source: Sci Adv. 2025 Apr 9;11(15):eadv2216. doi: 10.1126/sciadv.adv2216 (PMC11980845; doi:10.1126/sciadv.adv2216)
Supplement: Supplementary file 1 — Supplementary Text Figs. S1 to S30 Table S1 [file sciadv.adv2216_sm.pdf]

Science Advances
AAAS

Supplementary Materials for

Assessing depletion attractions between colloidal nanocrystals

Charles K. Ofosu *et al.*

Corresponding author: Delia J. Milliron, milliron@che.utexas.edu; Thomas M. Truskett, truskett@che.utexas.edu

Sci. Adv. **11**, eadv2216 (2025)
DOI: 10.1126/sciadv.adv2216

This PDF file includes:

Supplementary Text
Figs. S1 to S30
Table S1

1. Synthesis of Indium Oxide (In_2O_3) Nanocrystals (NCs)

1.1 Materials

All chemicals and reagents were purchased commercially and used as received without further purification. Indium(III) acetate (99.99%), oleic acid (90%, technical grade), oleyl alcohol (98%), and polystyrene of various molecular weights were purchased from Sigma-Aldrich and Polymer Source. Hexanes (99.8%), toluene (99.8%), isopropyl alcohol (95.0%) and ethanol (95.0%) were purchased from Fisher Chemical.

1.2 Synthesis

In_2O_3 NCs were synthesized on a Schlenk line by modifying a slow growth procedure. A precursor solution was prepared by adding 5.0 mmol of indium precursor (In(III) acetate) to 10 ml of oleic acid in a round bottom flask. The precursor solution was then put under vacuum and heated to 110°C. At this temperature, the precursor was purged with N_2 for 5 minutes, then degassed under vacuum for 15 minutes afterwards. This purging-degassing cycle was repeated three times. The precursor solution was then heated under N_2 to 150°C for 2 hours, allowing ligand exchange of oleate for acetate. While that exchange reaction proceeded, 13 ml of oleyl alcohol was put in a second round bottom flask, i .e. the reaction fl ask. The reaction flask was put under vacuum and, like the precursor solution, was heated to 110°C, followed by four cycles of 5-minute N_2 purge, and 15 minutes of degassing under vacuum. The oleyl alcohol was then heated to 290°C under N_2. Once the exchange reaction in the precursor flask was complete, the contents of the precursor flask were pulled into a syringe for slow injection into the reaction flask. The injection rate was set to 0.2 mL/min and the desired NC size dictated the volume of the precursor injected. Following the injection, the reaction flask was allowed to stay at 290°C for 20 min before being cooled to room temperature. This was to ensure the esterification runs

to completion at the reaction temperature. NCs were washed by 5 cycles of flocculating NCs with ethanol centrifuging at 7500 RPM, and redispersing in hexane. After the first wash, excess oleic acid was added to the dispersion (1–2% of the volume of the dispersion). The remaining 4 wash cycles were done until the supernatant was clear after centrifuging. The nanocrystals were finally redispersed in toluene once all washing steps are completed.

2. Instrumentation

2.1 Scanning Transmission Electron Microscopy (STEM)

Electron microscopy images were collected using a Hitachi S5500 scanning transmission electron microscope (STEM) with an accelerating voltage of 30 kV. To prepare the samples, a dispersion of oleate-capped In_2O_3 NCs in hexane was drop-cast onto Cu 400-mesh TEM grids with carbon support films. The samples were subsequently dried under vacuum before images were obtained.

2.2 Small-Angle X-ray Scattering (SAXS)

SAXS measurements were performed in transmission configuration with a sample-to-detector distance of 1.085 m on a SAXSLAB Ganesha instrument using Cu Kα radiation. Dilute NC dispersions were enclosed in flame-sealed glass capillaries (Charles-Supper Company, Boron Rich, 1.0 mm diameter, 0.01 mm wall thickness). A capillary containing neat solvent only was used for background subtraction. Scattering patterns were calibrated using a silver (I) behenate standard and were converted into 1D data by circular averaging using the Igor Pro-based Nika software for two-dimensional data reduction. Data were corrected for sample transmittance to extract the differential scattering cross section. The Irena tool suite for modeling and analysis in Igor Pro was used for background subtraction and form factor fitting. To determine the NC size, the corrected intensity profiles of the most dilute dispersions were fitted with the equation for the form factor of spherical particles with a Gaussian size distribution. The structure factors were determined by dividing the relatively concentrated NC dispersion scattering profiles by the spherical form factor fits of the most dilute dispersions, scaled to make the high wavenum-

ber of the structure factor normalized to 1. The primary peak positions in structure factors are identified by fitting split normal distributions near the peaks:

$$f(k, A, a_1, a_2, k_{\text{max}}) = \begin{cases} A \exp(-a_1(k - k_{\text{max}})^2), & k \leq k_{\text{max}} \\ A \exp(-a_2(k - k_{\text{max}})^2), & k > k_{\text{max}} \end{cases} \tag{S1}$$

2.3 Dynamic Light Scattering (DLS) and Zeta Potential

Hydrodynamic radii of the oleate-capped In_2O_3 NCs were measured by dynamic light scattering (DLS) with a Zetasizer Nano ZS equipped with a He-Ne laser ($\lambda = 633$ nm). Dilute NC dispersions in toluene ($\Phi = 0.0005$) were equilibrated to 25ºC. Second order intensity correlation functions ($g_{(2)}$) were extracted from the average of three consecutive measurements, each with 10 s acquisition time. The first-order field correlations ($g_{(1)}$) were subsequently calculated using from the intensity correlations using the relationship:

$$g_{(2)}(\tau) = B[1 + \beta g_{(1)}^2(\tau)] \tag{S2}$$

where τ is the correlation time. *B* and β are instrumental constants representing the baseline and coherence factor, respectively. The field correlation function can be represented as the correlation decay rate (Γ) from simple diffusive particles:

$$g_{(1)}(\tau) = e^{-\Gamma\tau} \tag{S3}$$

$$\Gamma = k^2 D_0 \tag{S4}$$

$$k = 4\pi n \frac{\sin(\theta/2)}{\lambda} \tag{S5}$$

where D_0 is the diffusion coefficient (dilute) and k is the wavenumber with scattering angle θ and wavelength λ. The experiments were performed in a back scattering configuration with $\theta = 175$º.

Particle size and dispersity were calculated by a least-square fitting of the first and second terms in the linear cumulant expansion of the initial logarithmic region of the field correlation curves. This can be expressed as

$$\ln\left(g_{(1)}(\tau)\right) = K_0 - \Gamma\tau + \frac{\mu}{2}\tau^2 \tag{S6}$$

where K_0, Γ, and μ are fitted parameters. The hydrodynamic radii of the particles were calculated from the spherical Stokes-Einstein approximation of the dilute diffusion coefficient (σ_{HS}) corresponding to the fitted decay rate (Γ):

$$D_0 = \frac{k_{\mathrm{B}}T}{6\pi\eta R_{\mathrm{H}}} \tag{S7}$$

where k_{B} is the Boltzmann constant, T is the solution temperature, and η is the dynamic viscosity. The polydispersity index (PDI) of the samples were estimated using the ratio of the second fitting term coefficient to the decay rate, $PDI = \mu/\Gamma$. The dynamic viscosity was corrected for polymer concentration according to methodology outlined by Kulicke and Kniewskie (*73*).

Polymer hydrodynamic radii were determined using similar methods but in a binary mixture of polymer and solvent in a dilute regime. All size results are averaged from at least 3 successive measurements.

The measurements of zeta potential (ZP) were also carried out using the Zetasizer Nano ZS on the dilute In_2O_3 NC dispersions in toluene ($\Phi = 0.0005$) equilibrated to 25 ° C. A dip cell was used for ZP measurements in the glass cuvette. The measurements were repeated at least three times.

3. Polymer Characterization

Linear polystyrene (PS) chains with molecular weights between $1.3 - 35$ kDa were obtained from Sigma-Aldrich and Polymer Source and used as received. This range was chosen to cover q from $\sim 0.15 - 1$. The polymers have a narrow size distribution, with a weight-average molecular weight to number-average molecular weight ratio ($M_{\mathrm{w}}/M_{\mathrm{n}}$) of less than 1.10. The intrinsic viscosity, $[\eta]^{\mathbf{tol}}$, was obtained from the experimental work of Fetters and coworkers and the radius of gyration, R_{g}, was calculated using the Flory-Fox equation:

$$R_{\mathrm{g}} = \left(\frac{[\eta]^{\mathbf{tol}} \cdot M_{\mathrm{w}}}{\Phi_0}\right)^{1/3} \tag{S8}$$

where Φ_0 is the Flory constant, 3.67×10^{24} mol^{-1}. The critical polymer overlap concentration, c^*, was calculated using the ideal volume of a polymer coil:

$$c^* = \frac{M_\mathrm{w}/N_\mathrm{A}}{\frac{4}{3}\pi R_\mathrm{g}^3} = \frac{\Phi_0}{\frac{4}{3}\pi N_\mathrm{A}[\eta]^\mathbf{tol}} \tag{S9}$$

where N_A is Avogadro's number. By relating contour length per monomer to molecular weight, we determined the contour length of each PS sample and subsequently calculated N_k, the number of Kuhn segments per contour length, with the Kuhn length for PS taken as 1.8 nm.

4. Theoretical Phase Diagrams

We modeled phase behavior of mixtures of NCs and non-absorbing polymer depletants using free volume theory (FVT) and generalized free volume theory (GFVT), where colloids are treated as hard spheres of diameter σ_HS. These theories are discussed extensively elsewhere (*4*,*26*). The colloidal osmotic pressure Π and chemical potential μ are non-dimensionalized as $\tilde{\Pi} = \Pi v_\mathrm{NC}/(k_\mathrm{B}T)$ and $\tilde{\mu} = \mu/(k_\mathrm{B}T)$, respectively, where $v_\mathrm{NC} = \pi\sigma_\mathrm{HS}^3/6$ is the thermodynamic volume of a single NC, $\Phi_\mathrm{NC} = \rho v_\mathrm{NC}$ is NC volume fraction, and ρ is NC number density. As described below, these intensive properties can be represented as sums of individual contributions from the hard-sphere repulsions and depletion attractions, $\tilde{\Pi} = \tilde{\Pi}^0 + \tilde{\Pi}^\mathrm{depl}$ and $\tilde{\mu} = \tilde{\mu}^0 + \tilde{\mu}^\mathrm{depl}$.

The osmotic pressure of the hard-sphere NC fluid is approximated by the Carnahan-Starling equation of state:

$$\tilde{\Pi}_f^0 = \frac{\Phi_\mathrm{NC} + \Phi_\mathrm{NC}^2 + \Phi_\mathrm{NC}^3 - \Phi_\mathrm{NC}^4}{(1-\Phi_\mathrm{NC})^3} \tag{S10}$$

where Φ_NC is the volume fraction of the NCs. The chemical potential of a hard-sphere fluid in this approximation is

$$\tilde{\mu}_f^0 = \ln\frac{\Lambda^3}{v_0} + \ln\Phi_\mathrm{NC} + \frac{3-\Phi_\mathrm{NC}}{(1-\Phi_\mathrm{NC})^3} - 3 \tag{S11}$$

where $\Lambda = h/\sqrt{2\pi m_\mathrm{NC} k_\mathrm{B} T}$ is the thermal de Broglie wavelength, with the NC mass m_NC and Planck constant h. The hard-sphere solid equation of state and chemical potential are approximated by the following relationships from the Lennard-Jones and Devonshire cell model,

respectively.

$$\tilde{\Pi}_s^0 = \frac{3\Phi_{\text{NC}}}{1-\Phi_{\text{NC}}/\Phi_{\text{cp}}} \tag{S12}$$

$$\tilde{\mu}_s^0 = \ln\frac{\Lambda^3}{v_{\text{NC}}} + \ln\left(\frac{27}{8\Phi_{\text{cp}}^3}\right) + 3\ln\left(\frac{\Phi_{\text{NC}}}{1-\Phi_{\text{NC}}/\Phi_{\text{cp}}}\right) + \frac{3\Phi_{\text{NC}}}{1-\Phi_{\text{NC}}/\Phi_{\text{cp}}} \tag{S13}$$

where $\Phi_{\text{cp}} \approx 0.74$ is the volume fraction of hard spheres at closed packing.

4.1 Free Volume Theory (FVT)

In FVT, the polymer depletants are simplified as penetrable hard spheres. The free-volume fraction α available to the polymer depletant is

$$\alpha = (1-\Phi_{\text{NC}})\exp[-Q(\Phi_{\text{NC}})] \tag{S14}$$

with

$$Q(\Phi_{\text{NC}}) = ay + by^2 + cy^3 \tag{S15}$$

$$y = \frac{\Phi_{\text{NC}}}{1-\Phi_{\text{NC}}} \tag{S16}$$

$$a = 3q + 3q^2 + q^3 \tag{S17}$$

$$b = \frac{9}{2}q^2 + 3q^3 \tag{S18}$$

$$c = 3q^3 \tag{S19}$$

The osmotic pressure of the depletant reservoir is given by Van 't Hoff's equation:

$$\tilde{\Pi}^R = \Phi_p^R q^{-3} \tag{S20}$$

where Φ_p^R is the volume fraction of a reservoir of polymer depletant in equilibrium with the depletant in the NC-depletant mixture. This volume fraction can be related to the polymer depletant concentration in the experiment by $c/c^* \approx \Phi_p = \alpha\Phi_p^R$. Non-dimensionalized NC chemical potential and osmotic pressure are expressed as sums of hard-sphere ($\tilde{\mu}^0$, $\tilde{\Pi}^0$) and plus depletion attraction ($\tilde{\mu}^{\text{depl}} = \tilde{\Pi}^R g(\Phi_{\text{NC}})$, $\tilde{\Pi}^{\text{depl}} = \tilde{\Pi}^R h(\Phi_{\text{NC}})$) contributions,

$$\tilde{\mu} = \tilde{\mu}^0 + \tilde{\Pi}^R g(\Phi_{\text{NC}}) \tag{S21}$$

$$\tilde{\Pi} = \tilde{\Pi}^0 + \tilde{\Pi}^R h(\Phi_{\text{NC}}) \tag{S22}$$

The functions $g(\Phi_{\text{NC}})$ and $h(\Phi_{\text{NC}})$ can be expressed

$$g(\Phi_{\text{NC}}) = \exp(-Q)[1 + (1+y)(a + 2by + 3cy^2)] \tag{S23}$$

$$h(\Phi_{\text{NC}}) = \exp(-Q)(1 + ay + 2by^2 + 3cy^3) \tag{S24}$$

From equations (S21) and (S22), the binodal line can be solved by equating pressures and chemical potentials,

$$\tilde{\Pi}^R = \frac{\tilde{\mu}_f^0(\Phi_l) - \tilde{\mu}_f^0(\Phi_g)}{g(\Phi_g) - g(\Phi_l)} = \frac{\tilde{\Pi}_f^0(\Phi_l) - \tilde{\Pi}_f^0(\Phi_g)}{h(\Phi_g) - h(\Phi_l)} \tag{S25}$$

where Φ_g and Φ_l are the coexisting colloid concentrations of the dilute ('gas') and the concentrated ('liquid') fluid phases, respectively. Given each Φ_g, we can find a unique corresponding Φ_l. The depletant volume fraction at the binodal line can be found by inserting Φ_g and Φ_l back into equation (S25) and equation (S20).

The spinodal line is defined by the stability condition

$$\left(\frac{\partial \tilde{\Pi}}{\partial \Phi_{\text{NC}}}\right)_T = 0 \tag{S26}$$

Therefore,

$$\frac{\partial \tilde{\Pi}^0}{\partial \Phi_{\text{NC}}} + \tilde{\Pi}^R \frac{\partial h(\Phi_{\text{NC}})}{\partial \Phi_{\text{NC}}} = 0 \tag{S27}$$

and

$$\Phi_{d,\text{spinodal}}^R = -q^3 \frac{\partial \tilde{\Pi}^0}{\partial \Phi_{\text{NC}}} \left(\frac{\partial h(\Phi_{\text{NC}})}{\partial \Phi_{\text{NC}}}\right)^{-1} \tag{S28}$$

Fluid-solid coexistence conditions can be determined by equating chemical potentials (and osmotic pressures) between the two phases, yielding

$$\tilde{\Pi}^R = \frac{\tilde{\mu}_s^0(\Phi_s) - \tilde{\mu}_f^0(\Phi_f)}{g(\Phi_f) - g(\Phi_s)} = \frac{\tilde{\Pi}_s^0(\Phi_s) - \tilde{\Pi}_f^0(\Phi_f)}{h(\Phi_f) - h(\Phi_s)} \tag{S29}$$

4.2 Generalized Free Volume Theory (GFVT)

GFVT is an extension of FVT that considers how polymeric depletant interactions modify the depletion layer thickness and osmotic pressure in colloid-polymer mixtures (*4*,*26*). Here, we

adopt the GFVT expression for polymer depletion layer thickness near a colloid in a good solvent (δ_s/R):

$$\frac{\delta_s}{R} = 0.865 \left(q/\sqrt{1 + 3.95(\Phi_p^R)^{1.54}} \right)^{0.88} \tag{S30}$$

Under good solvent conditions, the osmotic pressure of the polymer reservoir is

$$q^3 \frac{\partial \tilde{\Pi}^R}{\partial \Phi_p^R} = 1 + 3.73(\Phi_p^R)^{1.31} \tag{S31}$$

$$\tilde{\Pi}^R = q^{-3} \left[\Phi_p^R + 1.62(\Phi_p^R)^{2.31} \right] \tag{S32}$$

The equation of state from GFVT is given by

$$\tilde{\mu} = \tilde{\mu}^0 + \int_0^{\Phi_p^R} g(\Phi_{\text{NC}}, \Phi_p^{R'}) \left(\frac{\partial \tilde{\Pi}^R}{\partial \Phi_p^{R'}} \right) d\Phi_p^{R'} \tag{S33}$$

$$\tilde{\Pi} = \tilde{\Pi}^0 + \int_0^{\Phi_p^R} h(\Phi_{\text{NC}}, \Phi_p^{R'}) \left(\frac{\partial \tilde{\Pi}^R}{\partial \Phi_p^{R'}} \right) d\Phi_p^{R'} \tag{S34}$$

Here, $\alpha(\Phi_{\text{NC}}, \Phi_p^R)$, $g(\Phi_{\text{NC}}, \Phi_p^R)$, and $h(\Phi_{\text{NC}}, \Phi_p^R)$ are defined in equations (S14), (S23), and (S24) with

$$a = 3\left(\frac{\delta_s}{R}\right) + 3\left(\frac{\delta_s}{R}\right)^2 + \left(\frac{\delta_s}{R}\right)^3 \tag{S35}$$

$$b = \frac{9}{2}\left(\frac{\delta_s}{R}\right)^2 + 3\left(\frac{\delta_s}{R}\right)^3 \tag{S36}$$

$$c = 3\left(\frac{\delta_s}{R}\right)^3 \tag{S37}$$

Hence, the gas-liquid or fluid-solid binodal is computed by finding the coexisting NC volume fractions $\Phi_{\text{NC},i}$ in phase i and $\Phi_{\text{NC},j}$ in phase j satisfying

$$\tilde{\Pi}(\Phi_{\text{NC},i}, \Phi_p^R) = \tilde{\Pi}(\Phi_{\text{NC},j}, \Phi_p^R) \tag{S38}$$

$$\tilde{\mu}(\Phi_{\text{NC},i}, \Phi_p^R) = \tilde{\mu}(\Phi_{\text{NC},j}, \Phi_p^R) \tag{S39}$$

The polymer concentration Φ_p in each of the coexisting phases is related to the reservoir polymer concentration Φ_p^R with $\alpha\Phi_p^R = \Phi_p \approx c/c^*$.

5. Theoretical Second Osmotic Virial Coefficient B_2

The second osmotic virial coefficient B_2 of effective colloid-colloid interactions can be calculated from the potential of mean force $U(r)$:

$$B_2 = -2\pi \int_0^\infty (\exp[-\beta U(r) - 1])r^2 dr \tag{S40}$$

where r is the distance between the centers of the colloids. The AOV potential U_{AOV} is used to obtain B_2^{AOV}. The B_2 of hard spheres is given by

$$B_2^{\text{HS}} = \frac{2}{3}\pi\sigma_{\text{HS}}^3 \tag{S41}$$

The analytical B_2 from FVT is given by

$$B_2^{\text{FVT}} = 4 - \Phi_p^R \left(6 + \frac{15}{2}q + 3q^2 + \frac{q^3}{2}\right) \tag{S42}$$

To our knowledge, there is no analytical B_2 expression reported for GFVT. Therefore, the B_2 value is calculated by fitting the osmotic pressure $\tilde{\Pi}$ with a virial expansion in NC volume fraction Φ_{NC}

$$\tilde{\Pi} = \Phi_{\text{NC}} + \sum_{n=2}^{\infty} B_n \Phi_{\text{NC}}^n \tag{S43}$$

The value of B_2 converges after including 3 virial terms, with the fitting range of Φ from 0 to 0.05. The fitted B_2 values from FVT match the the ones from analytical expression (fig. S12), validating this approach. Consequently, we present the B_2 values obtained in this way from GFVT for further analysis.

6. Theoretical Structure Factors

We estimated theoretical structure factors for our NC-depletant systems via Brownian dynamics (BD) simulations using HOOMD-blue (*75*) and by numerically solving the Ornstein-Zernike equation from integral equation theory with an appropriate closure via the pyPRISM package (*74*). To check the closure accuracy, we cross-validated structure factors derived from integral equation theory with those from BD simulations.

6.1 Brownian Dynamics Simulations

We simulated $N = 10^4$ identical colloidal spheres in a periodically replicated cell using HOOMD-blue. Colloid-colloid interactions were modeled using the effective AOV pair potential

$$U_{\mathrm{AOV}}(r) = \begin{cases} U_{\mathrm{HS}}(r), & r < \sigma_{\mathrm{HS}} \\ -\Phi_p^R(1+q^{-1})^3\left[1 - \dfrac{3r}{2\sigma_{\mathrm{HS}}(1+q)} + \dfrac{r^3}{8\sigma_{\mathrm{HS}}^3(1+q)^3}\right], & \sigma_{\mathrm{HS}} \le r \le \sigma_{\mathrm{HS}} + 2R_{\mathrm{g}} \\ 0, & r > \sigma_{\mathrm{HS}} + 2R_{\mathrm{g}} \end{cases} \tag{S44}$$

where $\Phi_p^R = \Phi_p = c/c^*$ and $q = 2R_{\mathrm{g}}/\sigma_{\mathrm{HS}}$ were set to the experimental values. The interaction range reflects where depletion layers for the two colloids begin to overlap. For BD simulations, U_{HS} was approximated using the Heyes-Melrose potential (*77*), $U_{\mathrm{HS,BD}}$:

$$U_{\mathrm{HS,BD}}(r) = \begin{cases} \dfrac{(\sigma_{\mathrm{HS}} - r)^2}{4\Delta t}, & r < \sigma_{\mathrm{HS}} \\ 0, & r \ge \sigma_{\mathrm{HS}} \end{cases} \tag{S45}$$

where σ_{HS} is the colloid diameter and Δt is the time elapsed between time steps. The colloid diameter was chosen to be the characteristic length scale (unity) of the simulation, and the time elapsed between steps was set to 10^{-6} in HOOMD-blue's default nondimensional time units. This potential behaves like a hard-sphere interaction in BD simulations by shifting any newly overlapping spheres into contact on the very next time step irrespective of the chosen value of Δt (*77*).

The simulation cell was sized to match the colloid volume fraction, Φ_{NC}. Particles were thermally pre-equilibrated as hard spheres (no implicit polymer) first before turning on the AOV attractive potential for a 10^7 time step equilibration period. The simulation dynamics were then run for a final 10^7 time steps while sampling frames at a rate of one every 10^5 time steps. Finally, the structure factor was calculated for each sampled frame and averaged using the direct static structure factor method from the Freud trajectory analysis package (*76*). This process was replicated five times using different seeds and the results averaged for each state point. The resulting structure factors were in good agreement with those previously reported in literature (*40*).

6.2 Integral Equation Theory (IET)

The Ornstein-Zernike equation from IET of the liquid state was solved using the pyPRISM python package for both an implicit-depletant, one-component system and an explicit-depletant, two-component system. The implicit-depletant, one-component system accounted for the polymer by using the AOV potential between colloids and is hence referred to as the AOV model in the main text. The explicit-depletant, two-component system treats the polymer as penetrable hard spheres of diameter, $2R_{\mathrm{g}}$, meaning they interact with the colloids as hard spheres, but have no interaction potential among themselves. In both cases, the hypernetted chain closure was used to calculate the colloid-colloid direct correlation function. In the explicit depletant system, the Percus-Yevick closure was used in calculating both the colloid-depletant and depletant-depletant direct correlation function. All of the requisite closures and potentials were readily available in the pyPRISM package with the exception of the AOV potential. To model this potential, we implemented a tabulated potential class identical to that used in the BD simulation but with $U_{\mathrm{HS}} = \mathrm{C}$, where C is a sufficiently large value to mimic an infinitely steep potential. This approximation for U_{HS} is the standard convention set in the pyPRISM package to avoid numerical errors associated with an infinite potential while preserving hard-sphere-like behavior (*74*).

The implicit-depletant BD simulation structure factors and both the implicit- and explicit-depletant structure factors from IET are all in good agreement with each other (as seen in figs. S16-S19), leading to excellent confidence in the accuracy of the re sults. We do note that the structure factors derived from IET deviate from the simulated structure factors at low k and high depletion strength. This is likely due to the breakdown of the HNC theory in predicting long-range trends and in its approximation to the direct correlation function of the attractive depletion tail from the AOV potential (*74*). Regardless, the deviation is not significant in most cases, so we only directly compare experimental structure factors to those calculated using the implicit-depletant IET (or AOV model) in both the main text and SI (see figs. S 21-S30). For strong interparticle attractions, especially near the spinodal, IET can encounter convergence

issues. For any panel where the IET structure factor is omitted, a converged IET solution could not be obtained. Finally, we tested a few explicit-depletant systems where we set an inter-depletant HS potential with an interaction range $\lambda q \sigma_{\text{HS}}$ and λ varying between 0 and 1, leaving the depletant-colloid interaction range unaltered. The results (fig. S15) show that more repulsive depletant-depletant interactions lead to colloid-colloid structure factors that manifest strengthened attractive depletion attractions.

7. Supporting Figures and Tables

7.1 Scanning Transmission Electron Microscopy

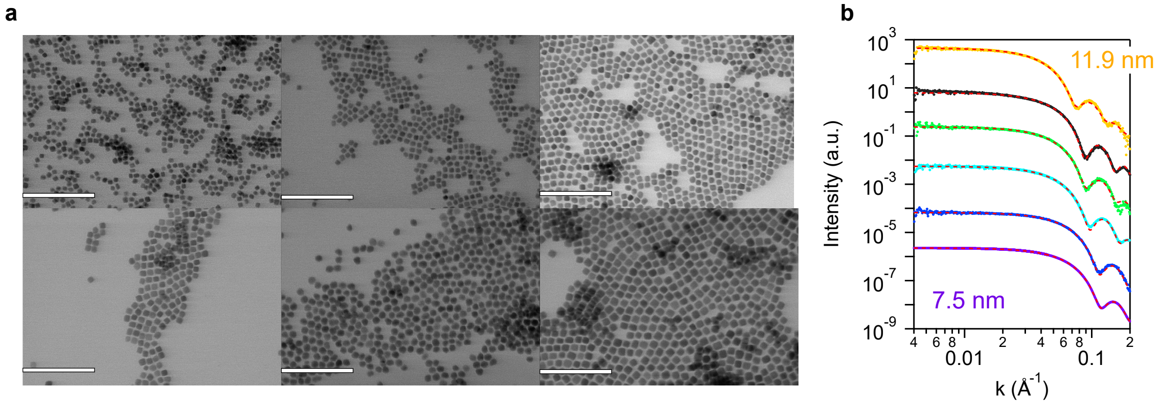

fig. S1. Confirmation of quasi-spherical morphology, size, and size distribution of the NCs **(a)** STEM images of the NCs used in this study ranging from 7.5 nm to 11.9 nm. Scale bar = 100 nm. **(b)** Form factors [P(k)] of NCs used in this study. Dotted lines represent fits to obtain size and size distribution of the NCs.

7.2 SAXS Intensities and Structure Factors of In_2O_3 NC in Toluene (without polymer)

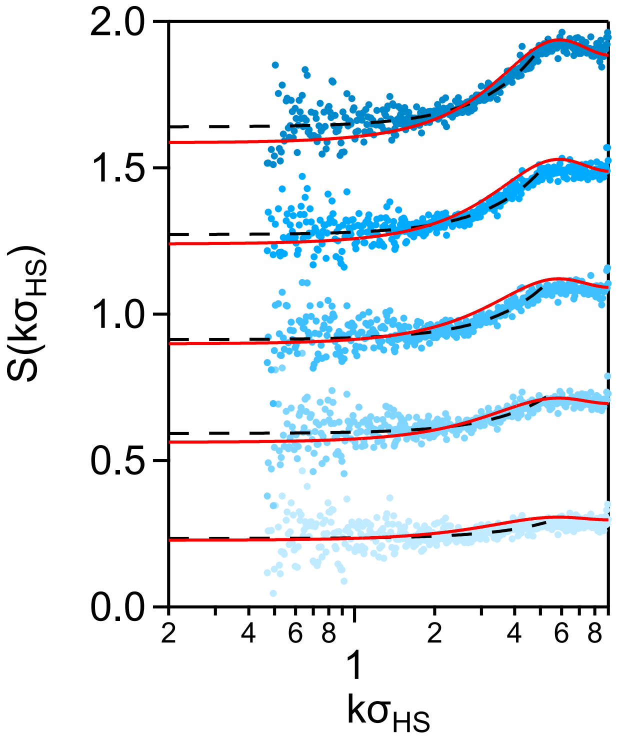

fig. S2. NC structure factors $S(k)$ at a NC core volume fractions $0.005 \leq \Phi \leq 0.025$, vertically shifted for clarity. Solid red curves represent the exact solution of the Percus-Yevick integral equation for the hard-sphere fluid at a packing fraction consistent with the experimental NC number density ρ and a hard-sphere diameter $\sigma_{\mathrm{HS}} = 11.8$ nm obtained from the experimental second osmotic virial coefficient (B_2^{HS}) analysis.

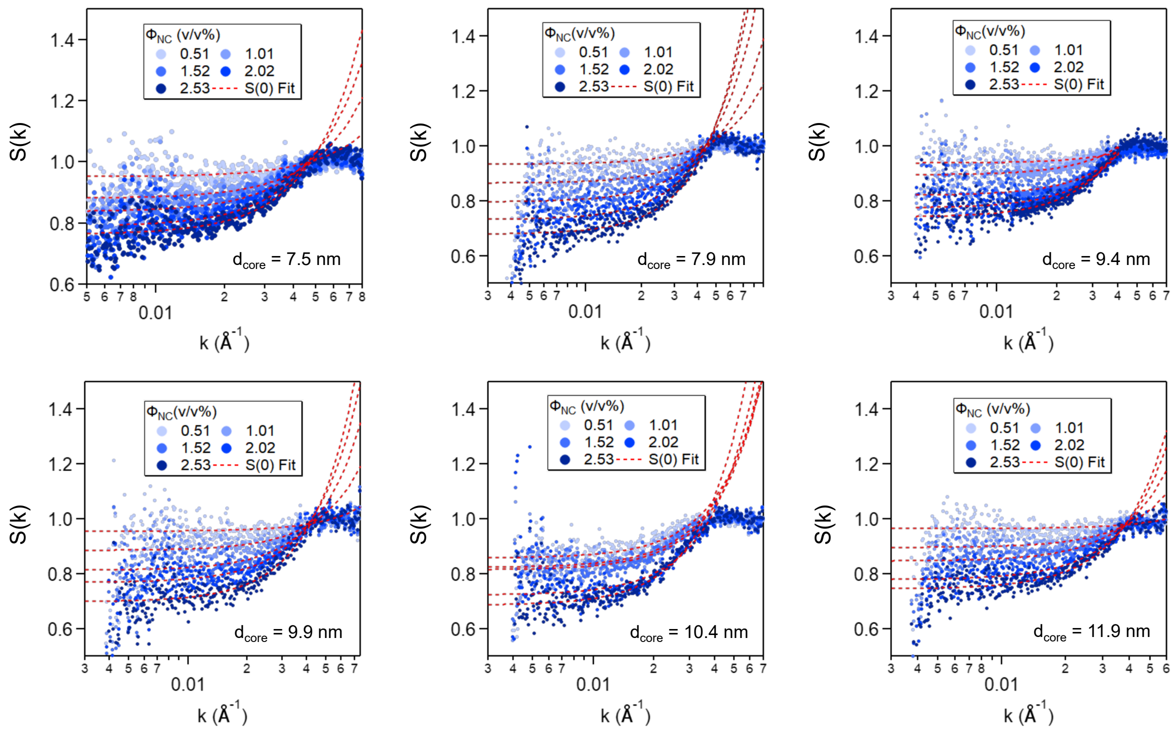

fig. S3. Determining effective hard-sphere diameter of NCs without polymer added (a-f) Colloid-colloid structure factors of In_2O_3 NCs in toluene without polymer added with core diameters 7.5 nm, 7.9 nm, 9.4 nm, 9.9 nm, 10.4 nm, and 11.9 nm. Red curves represent quadratic fits to the low k data used to extract $S(0)$.

7.3 Extraction of Second Osmotic Virial Coefficient B_2

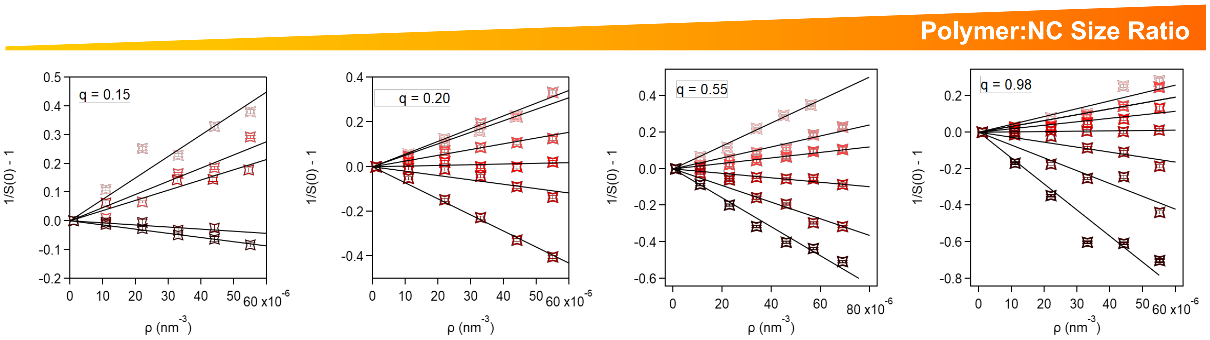

fig. S4. Extracting B_2 $1/S(0) - 1$ versus ρ (nm^{-3}) for varying c/c^* at $0.15 \leq q \leq 0.98$ obtained from increasing PS M_w at a hard-sphere diameter $\sigma_{\mathrm{HS}} = 11.8$ nm. Progression from light to dark red indicate increasing c/c^*, and increasing attraction. Black lines are linear fits to data with slope $2B_2$.

7.4 Theoretical Phase Diagrams

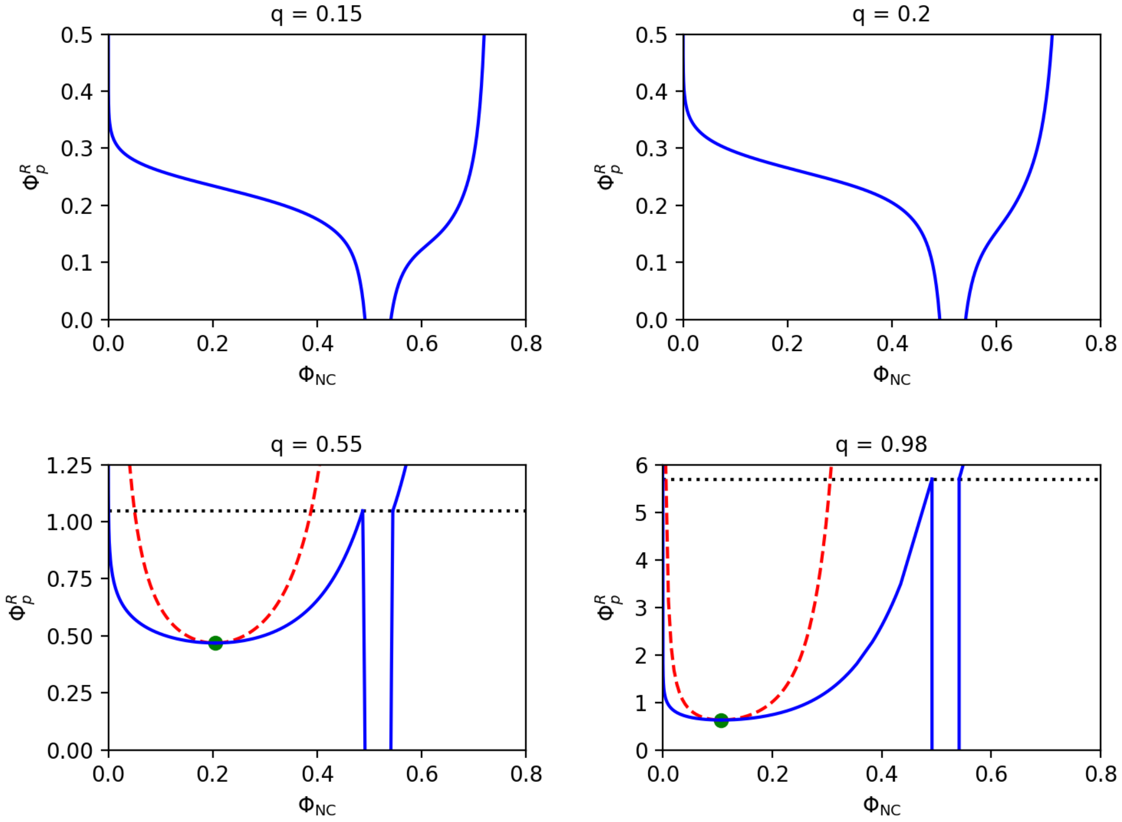

fig. S5. Phase diagrams from FVT at various size ratio q. Black dot line: triple point. Green dot: critical point. Blue line: binodal lines. Red dash line: spinodal line.

7.5 SAXS Intensities and Structure Factors of In_2O_3 NC–polystyrene mixtures

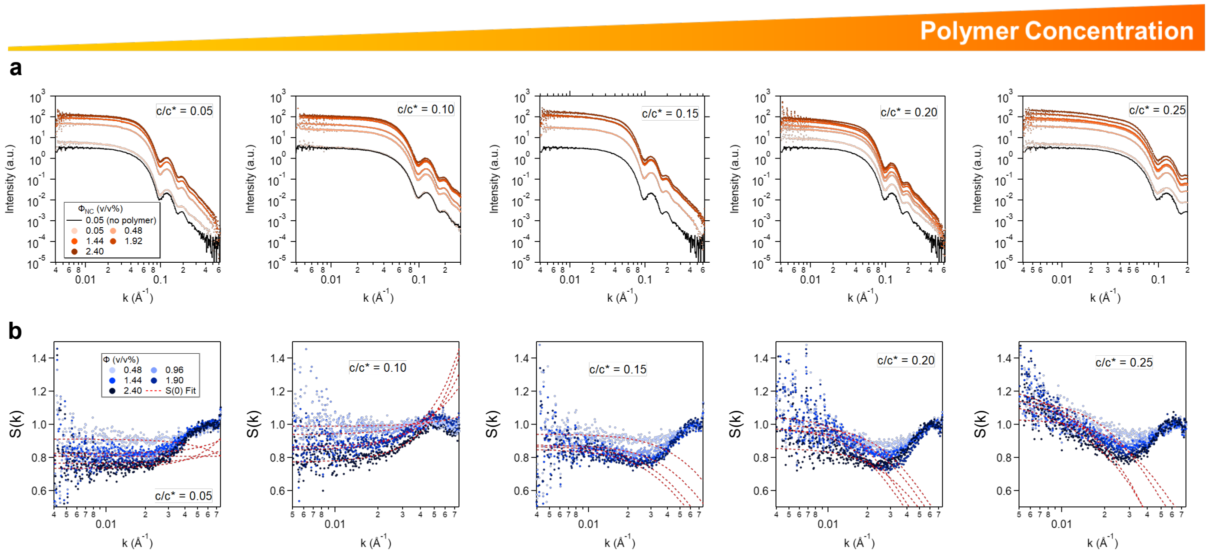

fig. S6. Scattering intensities and colloid-colloid structure factors at $q = 0.15$ **(**$\sigma_{\mathbf{HS}}$ **= 11.8 nm,** $M_{\mathbf{w}}$ **(PS) = 1.3 kDa) (a)** SAXS intensities of the nanocrystal-polymer mixtures, with each plot showing the scattering intensity progression at increasing Φ (lighter to darker orange). c/c^* increases from left to right plots towards stable phase limit. **(b)** Colloid-colloid structure factors $S(k)$ at increasing Φ (light to dark blue), with increasing c/c^* from left to right as in (a). Red curves represent Lorentzian fits to extract $S(0)$ values for B_2 analysis.

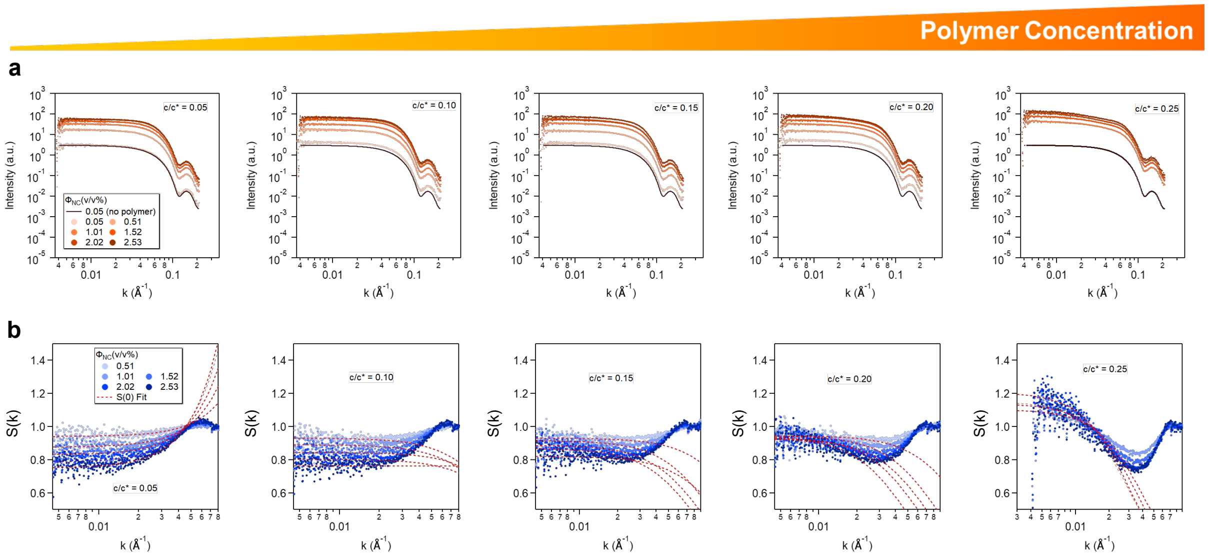

fig. S7. Scattering intensities and colloid-colloid structure factors at $q = 0.17$ **(**$\sigma_{\mathbf{HS}}$ **= 10.3 nm,** $M_{\mathbf{w}}$ **(PS) = 1.3 kDa) (a)** SAXS intensities of the nanocrystal-polymer mixtures, with each plot showing the scattering intensity progression at increasing Φ (lighter to darker orange). c/c^* increases from left to right plots towards stable phase limit. **(b)** Colloid-colloid structure factors $S(k)$ at increasing Φ (light to dark blue), with increasing c/c^* from left to right as in (a). Red curves represent Lorentzian fits to extract $S(0)$ values for B_2 analysis.

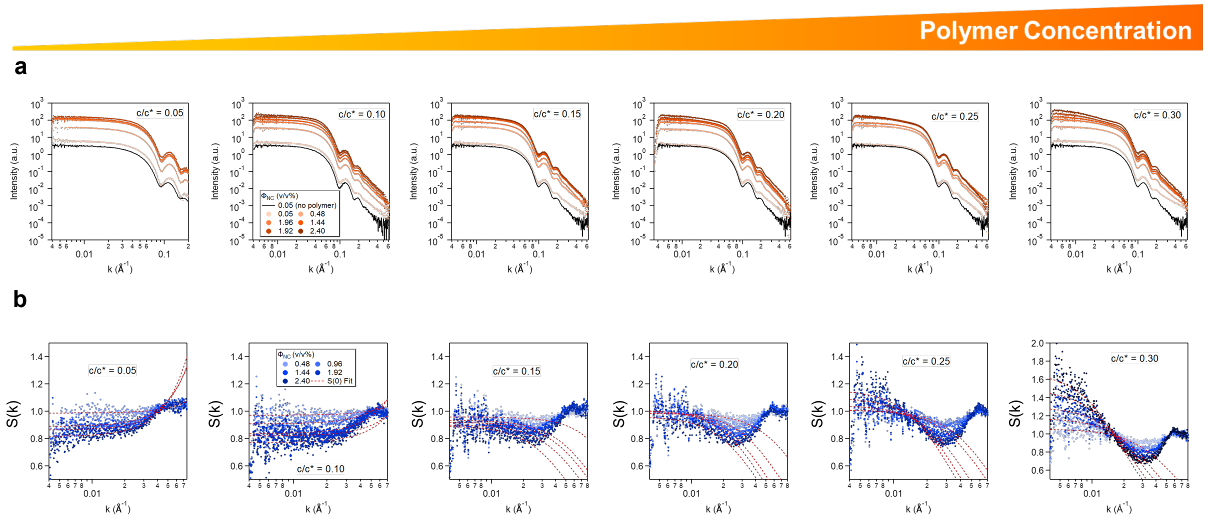

fig. S8. Scattering intensities and colloid-colloid structure factors at $q = 0.20$ **(**$\sigma_{\mathbf{HS}}$ **= 11.8 nm,** $M_{\mathbf{w}}$ **(PS) = 2.2 kDa) (a)** SAXS intensities of the nanocrystal-polymer mixtures, with each plot showing the scattering intensity progression at increasing Φ (lighter to darker orange). c/c^* increases from left to right plots towards stable phase limit. **(b)** Colloid-colloid structure factors $S(k)$ at increasing Φ (light to dark blue), with increasing c/c^* from left to right as in (a). Red curves represent Lorentzian fits to extract $S(0)$ values for B_2 analysis.

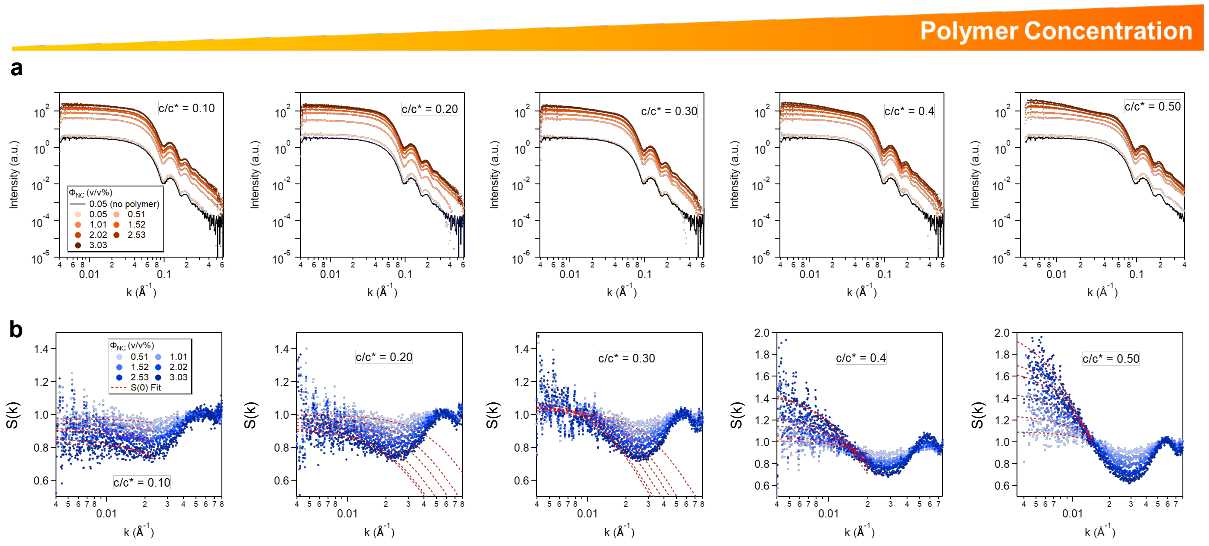

fig. S9. Scattering intensities and colloid-colloid structure factors at $q = 0.55$ **(**$\sigma_{\mathbf{HS}}$ **= 11.8 nm,** $M_{\mathbf{w}}$ **(PS) = 13 kDa) (a)** SAXS intensities of the nanocrystal-polymer mixtures, with each plot showing the scattering intensity progression at increasing Φ (lighter to darker orange). c/c^{*} increases from left to right plots towards stable phase limit. **(b)** Colloid-colloid structure factors $S(k)$ at increasing Φ (light to dark blue), with increasing c/c^{*} from left to right as in (a). Red curves represent Lorentzian fits to extract $S(0)$ values for B_2 analysis.

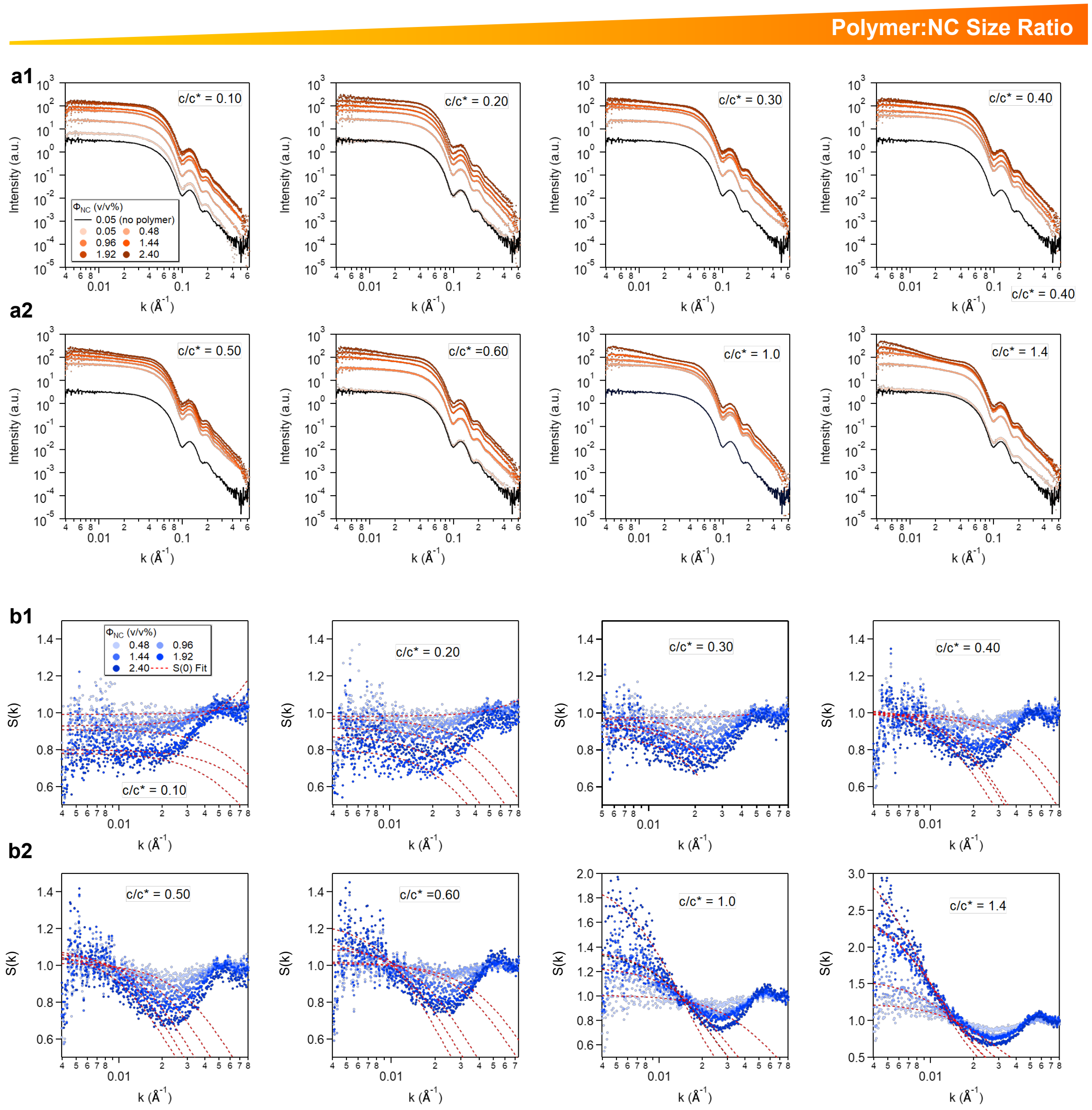

fig. S10. Scattering intensities and colloid-colloid structure factors at $q = 0.98$ **(**$\sigma_{\mathbf{HS}}$ **= 11.8 nm,** M_w **(PS) = 35 kDa) (a)** SAXS intensities of the nanocrystal-polymer mixtures, with each plot showing the scattering intensity progression at increasing Φ (lighter to darker orange). c/c^* increases from left to right plots towards stable phase limit. **(b)** Colloid-colloid structure factors $S(k)$ at increasing Φ (light to dark blue), with increasing c/c^* from left to right as in (a). Red curves represent Lorentzian fits to extract $S(0)$ values for B_2 analysis.

7.6 Second Osmotic Virial Coefficient B_2 - Theory and Experiment

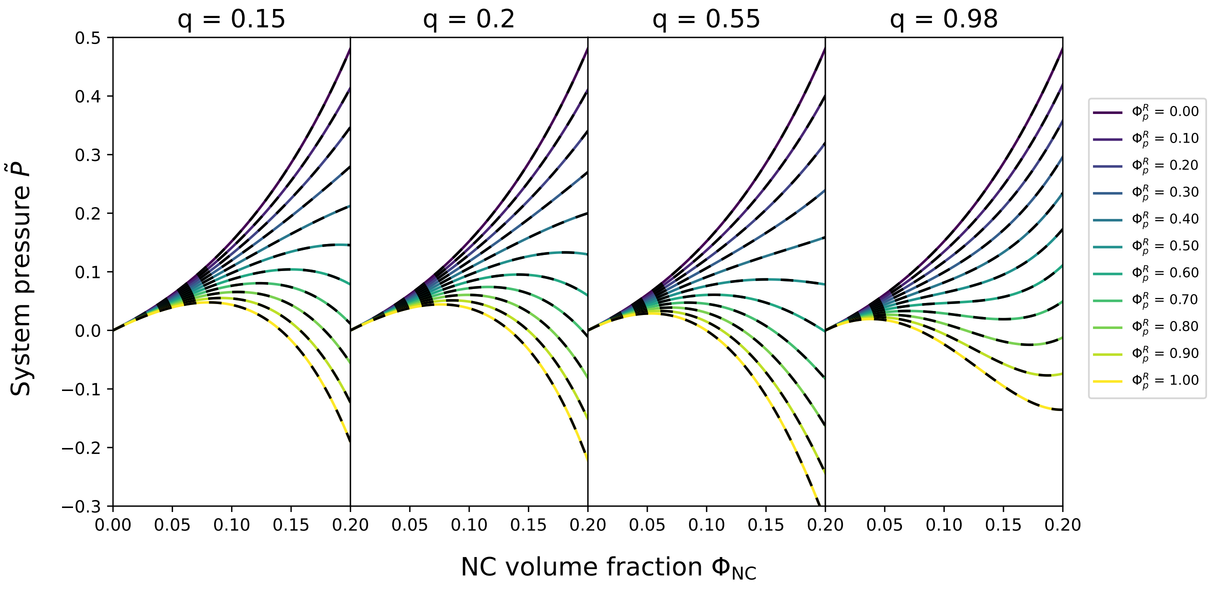

fig. S11. Virial coefficient fit of pressure from FVT at various size ratio q

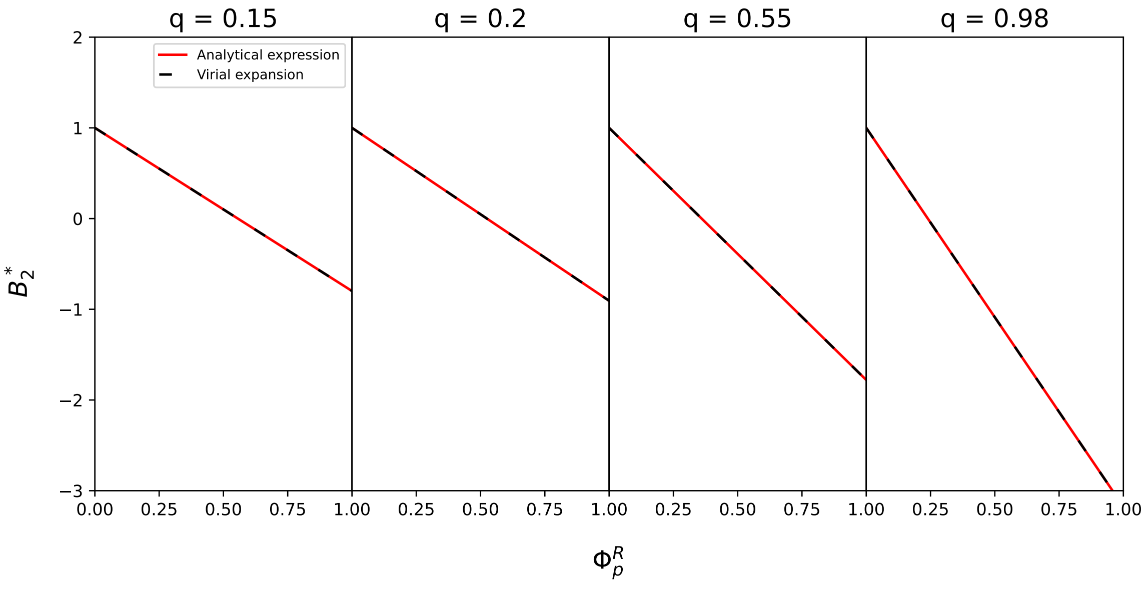

fig. S12. Comparison of FVT B_2 from the analytical expression and virial coefficient fit at various size ratio q. The solid lines represent the analytical expression from equation (S42), whereas the black dashed lines represent the results from the virial coefficient fits.

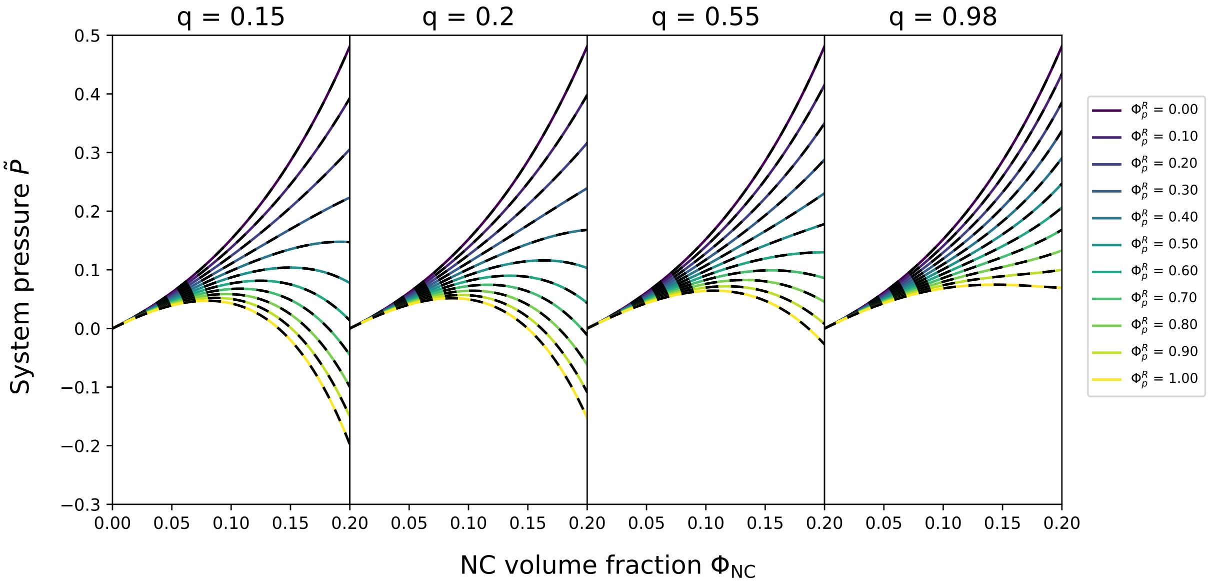

fig. S13. Virial coefficient fit of pressure from GFVT at various size ratio q. The solid lines are the GFVT pressure, and the black dashed lines represent the results from the virial coefficient fits.

7.7 Small Polymer Effect on the Pairwise Interactions

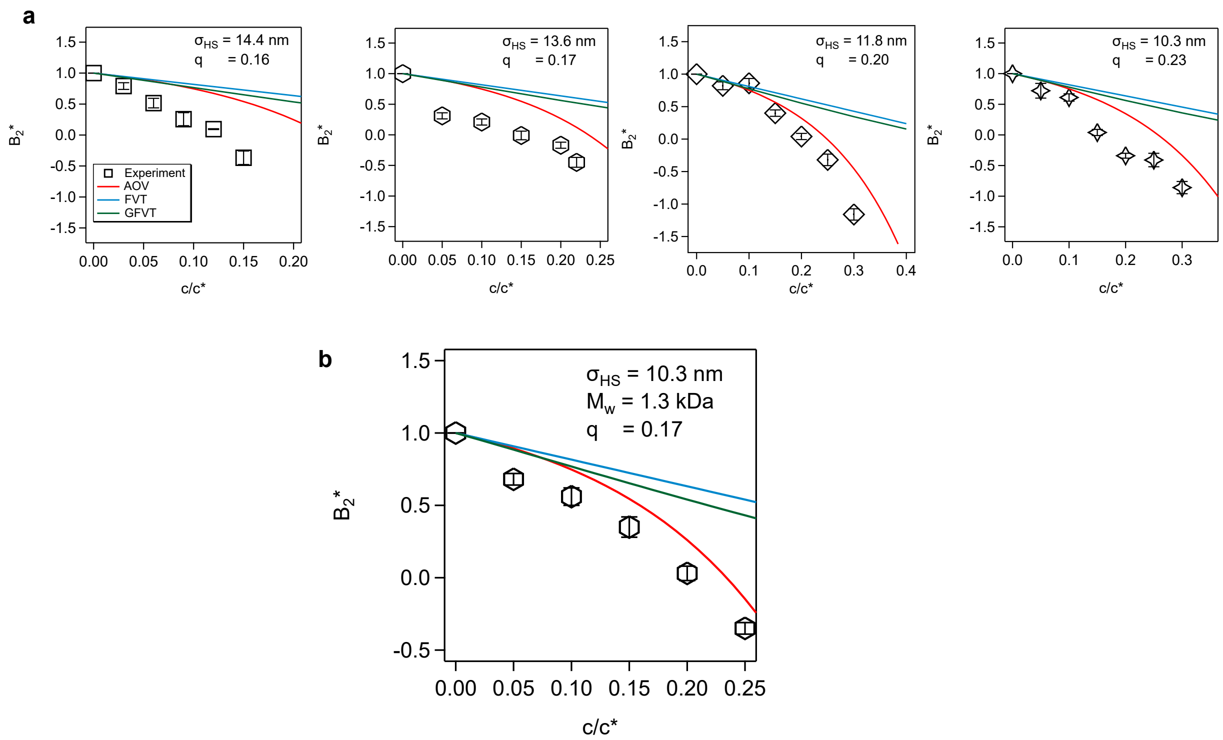

fig. S14. B_2^* vs c/c^* relationship at (a) $0.16 \leq q \leq 0.23$ and where the increase in q is achieved via decreasing σ_{HS} at a constant R_{g} ($R_{\mathrm{g}} = 1.2$ nm, $M_{\mathrm{w}} = 2.2$ kDa) and (b) when $R_{\mathrm{g}} = 0.89$ nm, $M_{\mathrm{w}} = 1.3$ kDa. Included on the plots are the AOV, FVT, and GFVT model predictions of the $B_2^* - c/c^*$ relationship, demonstrating the limits of applicability of each theory.

7.8 Structure Factors–Simulation and Integral Equation Theory Results

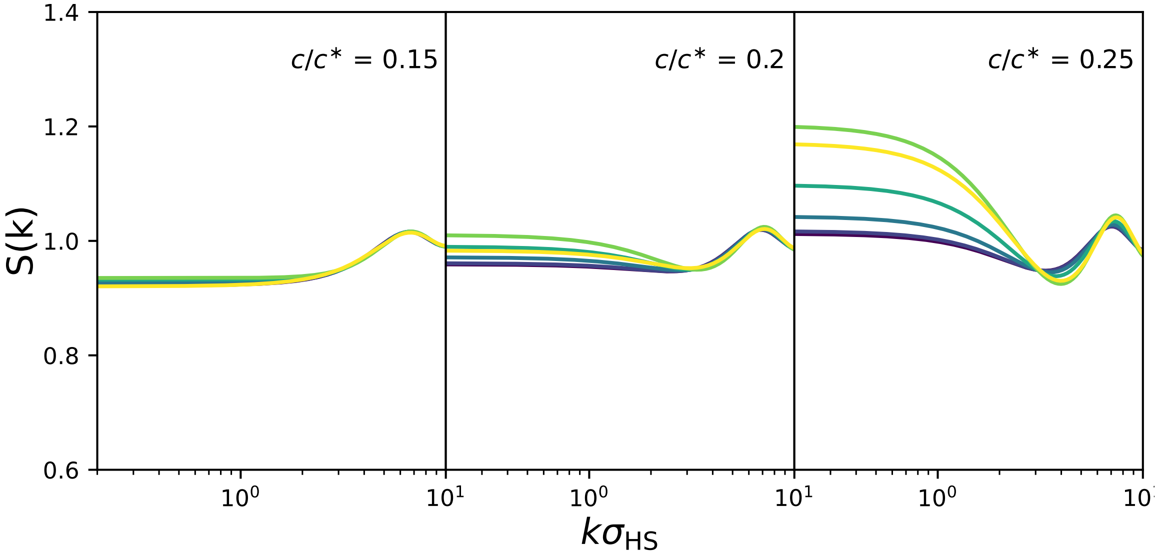

fig. S15. Structure factors from HS-depletant IET calculations for q = 0.2, $\Phi_{NC} = 0.019$, and λ ranges from 0.0 (purple) to 1.0 (yellow). More repulsive depletant-depletant interactions yield structure factors consistent with stronger depletion attractions.

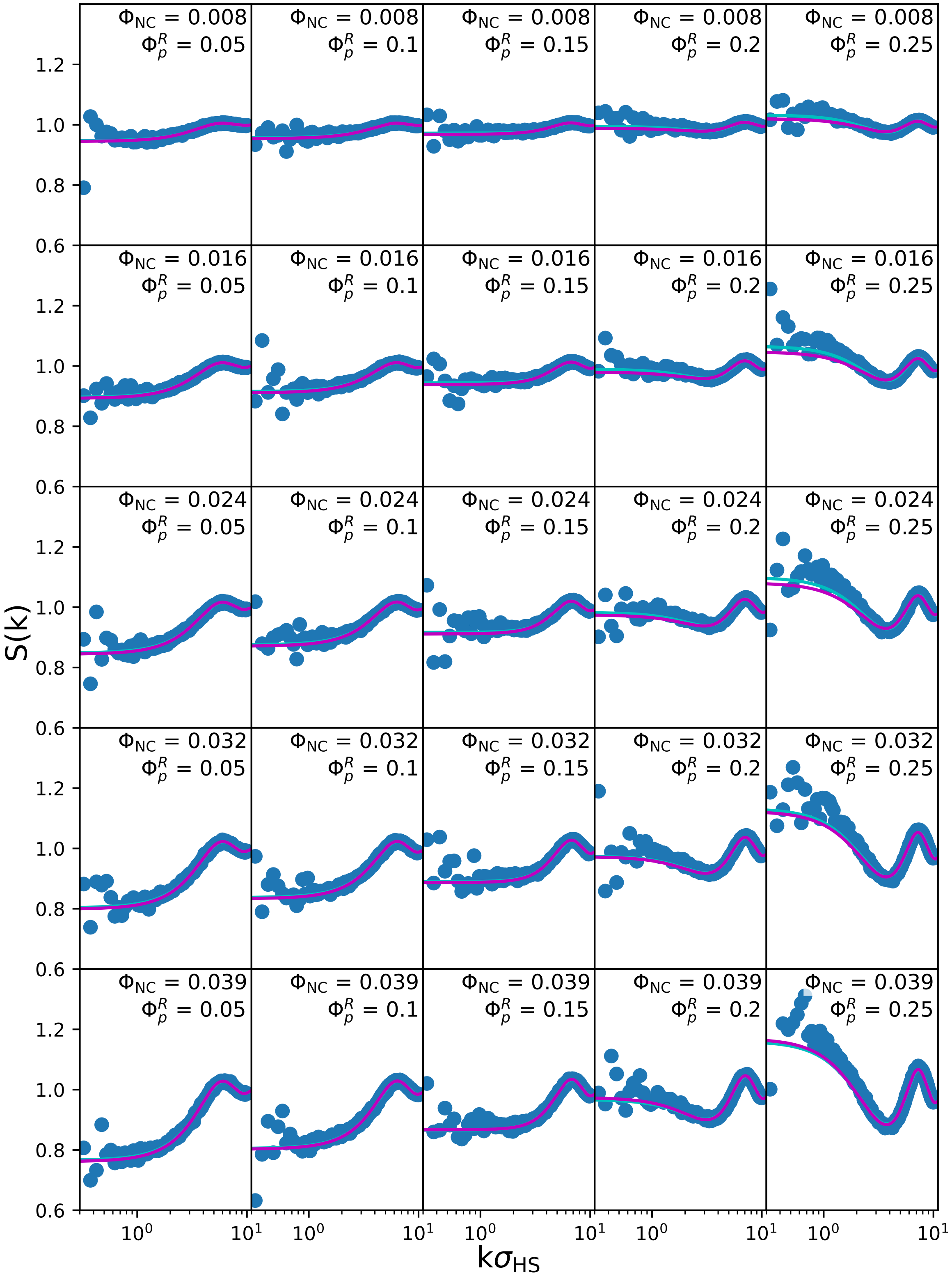

fig. S16. Structure factors from AOV simulations (blue dots), AOV IET (cyan line), PHS IET (magenta line) at q = 0.15 and the Φ_{NC} and Φ_p^R indicated.

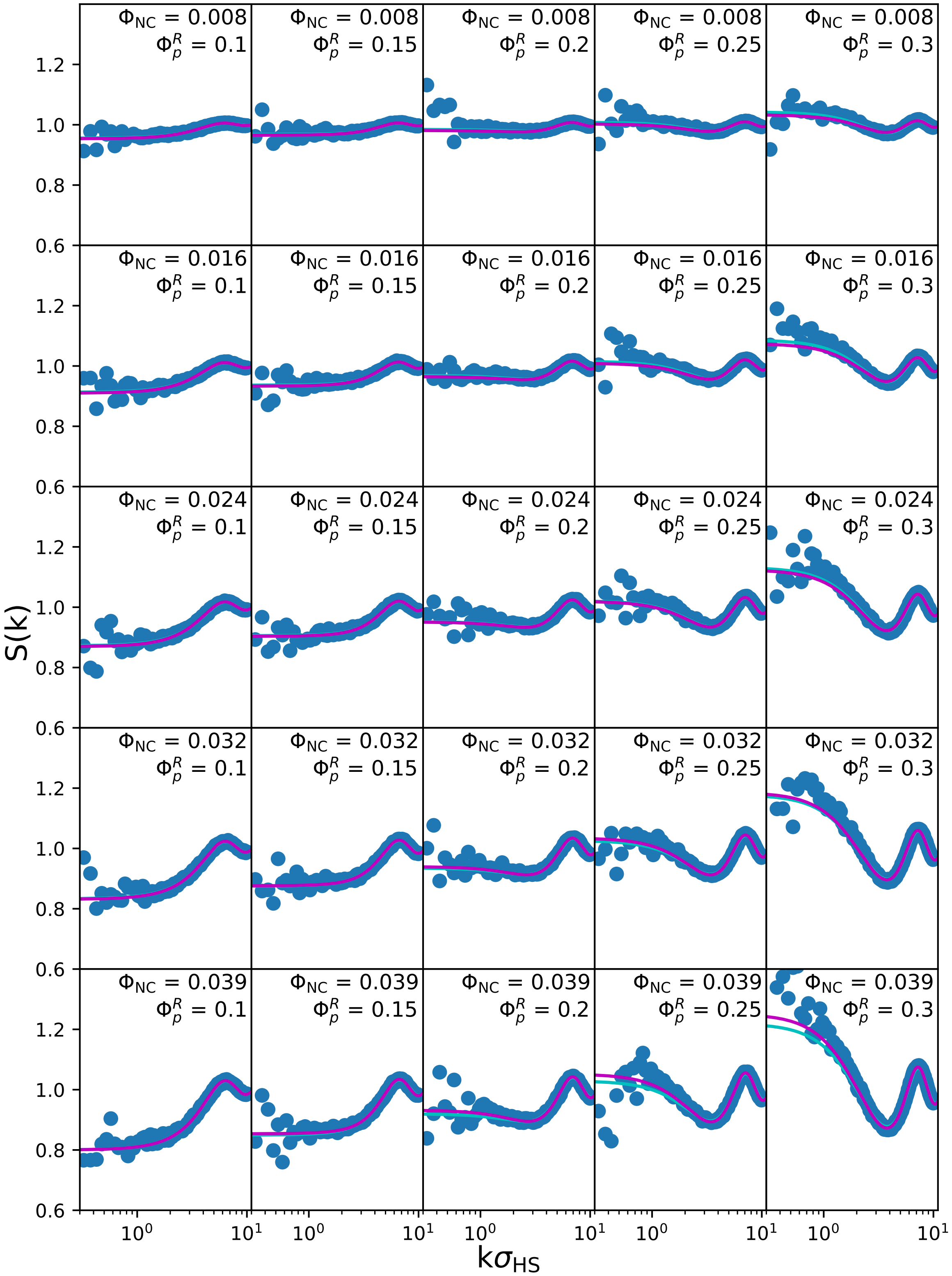

fig. S17. Structure factors from AOV simulations (blue dots), AOV IET (cyan line), PHS IET (magenta line) at q = 0.2 and the $\Phi_{\rm NC}$ and Φ_p^R indicated.

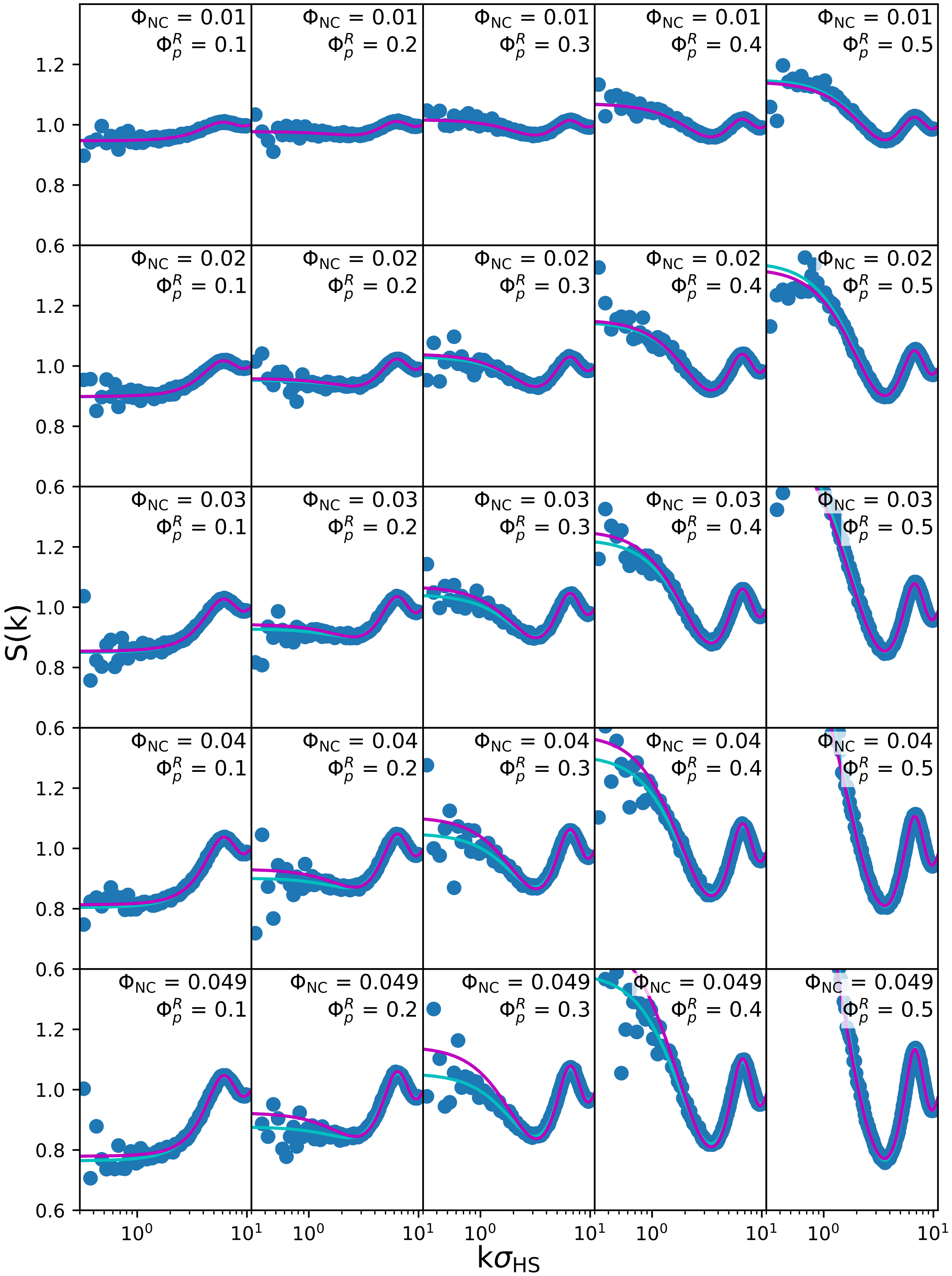

fig. S18. Structure factors from AOV simulations (blue dots), AOV IET (cyan line), PHS IET (magenta line) at q = 0.55 and the Φ_{NC} and Φ_p^R indicated.

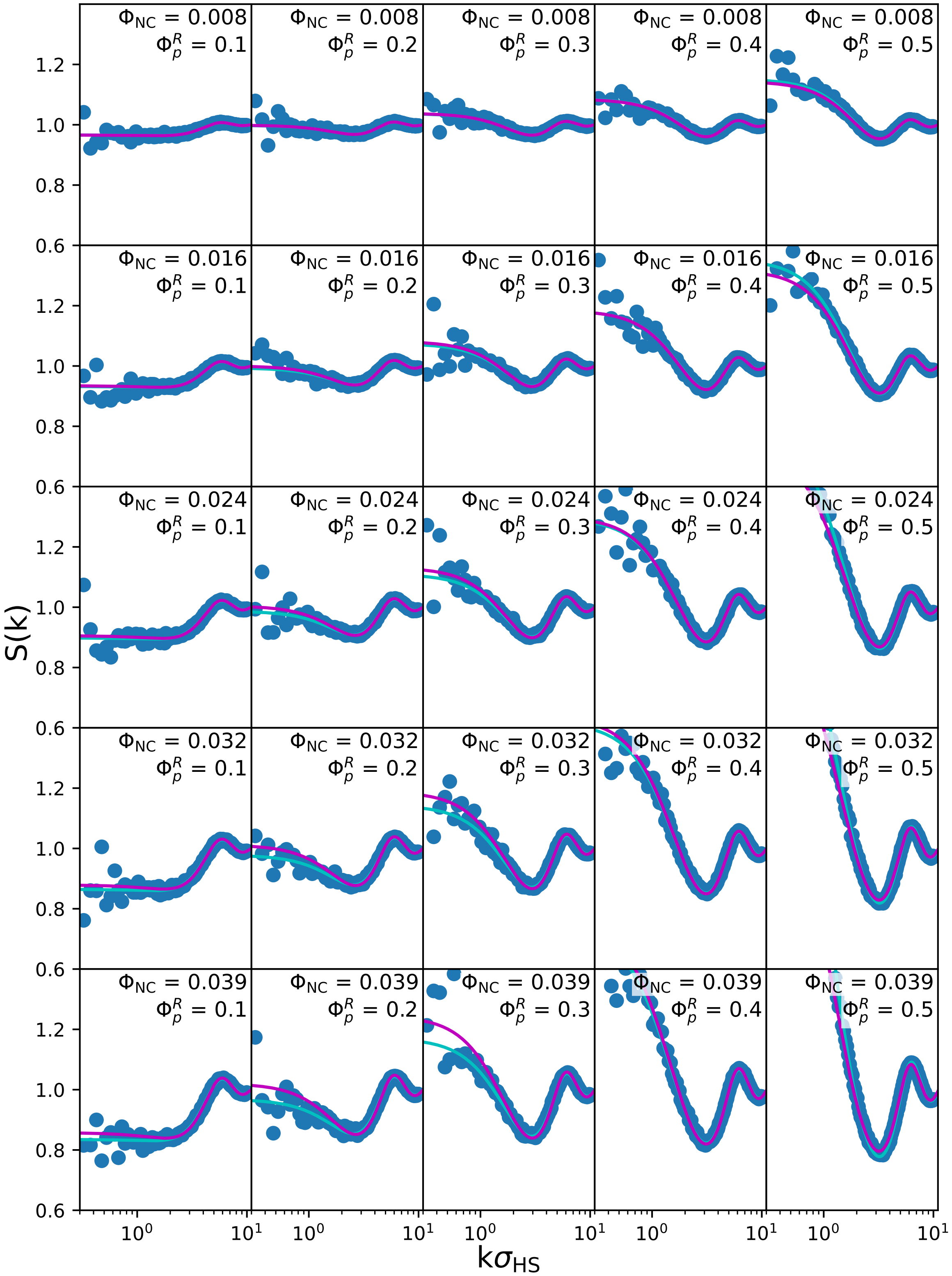

fig. S19. Structure factors from AOV simulations (blue dots), AOV IET (cyan line), PHS IET (magenta line) at q = 0.98 and the Φ_{NC} and Φ_p^R indicated.

7.9 Dynamic Light Scattering and Zeta Potential Results

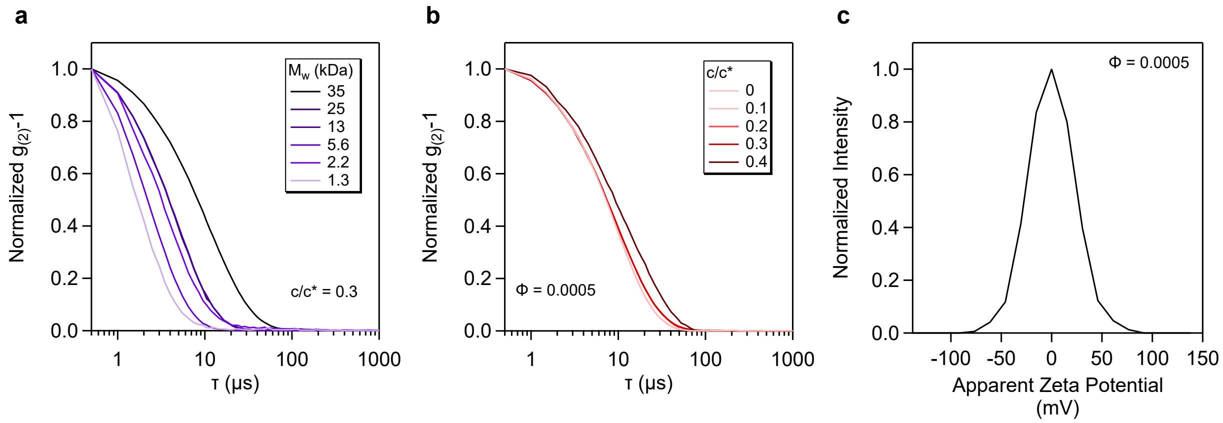

fig. S20. Characterizing polystyrene and dispersed NCs in toluene (a) Normalized intensity correlation functions ($g_{(2)} - 1$) vs lag time (τ) of different molecular weight polystyrene in toluene, without nanocrystals. Polymer concentration was kept constant at $c/c^* = 0.3$, chosen to stay below overlap while allowing for sufficient light scattering. **(b)** Normalized intensity correlation functions ($g_{(2)} - 1$) vs lag time (τ) of dilute ($\Phi = 0.0005$) NCs dispersed in 13k Da polystyrene solutions of different concentrations $0 \leq c/c^* \leq 0.4$. **(c)** Zeta potential measurement of oleate-capped In_2O_3 NCs ($\Phi = 0.005$) in toluene indicating no surface charge.

7.10 Structure Factors–Integral Equation Theory Fits to Experimental Data

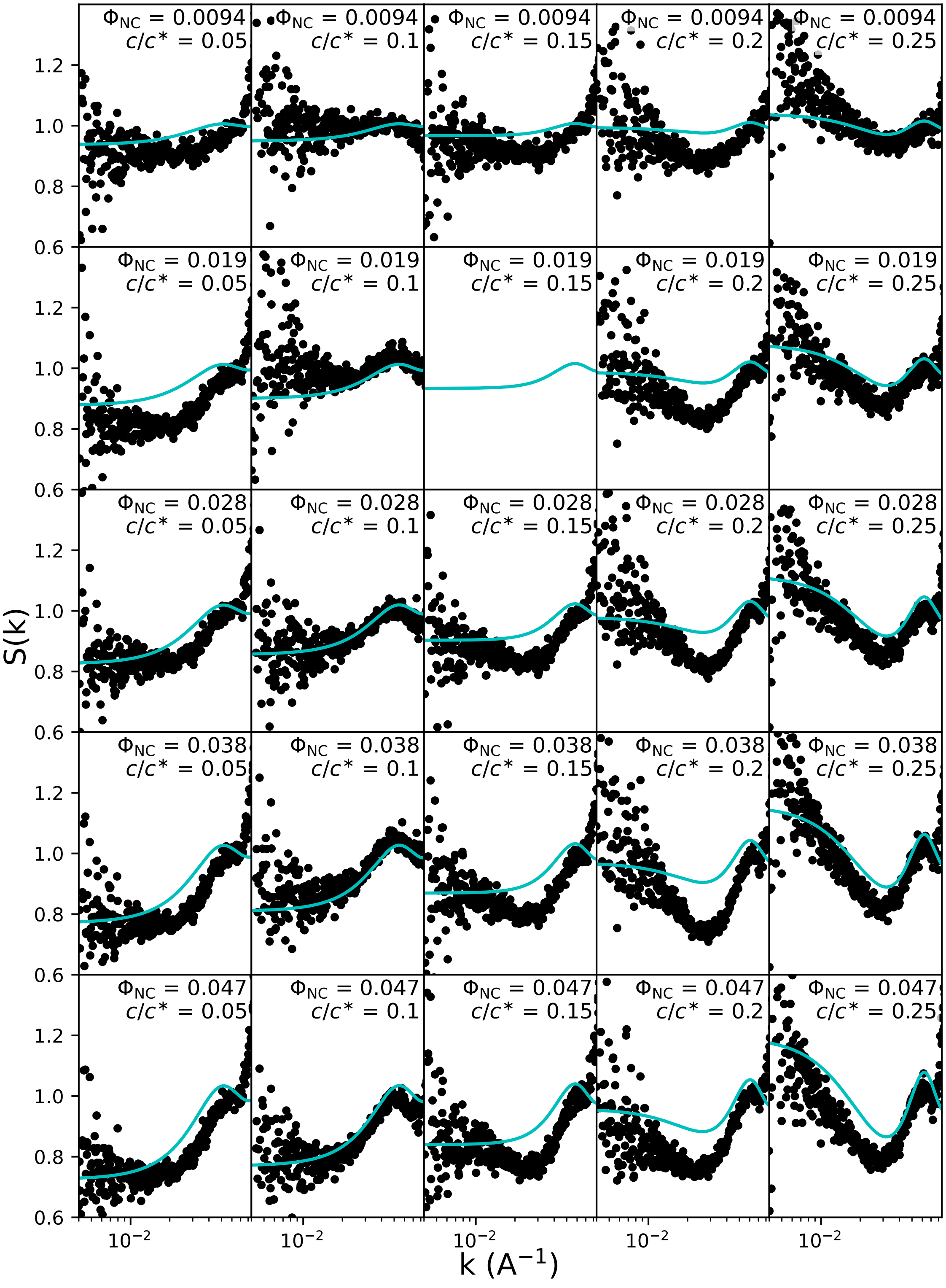

fig. S21. Structure factors from experiment (black dots) and AOV IET (cyan line) at q = 0.15 (σ_{HS} = 11.8 nm, polymer M_w = 1.3 kDa) and the Φ_{NC} and c/c^* indicated.

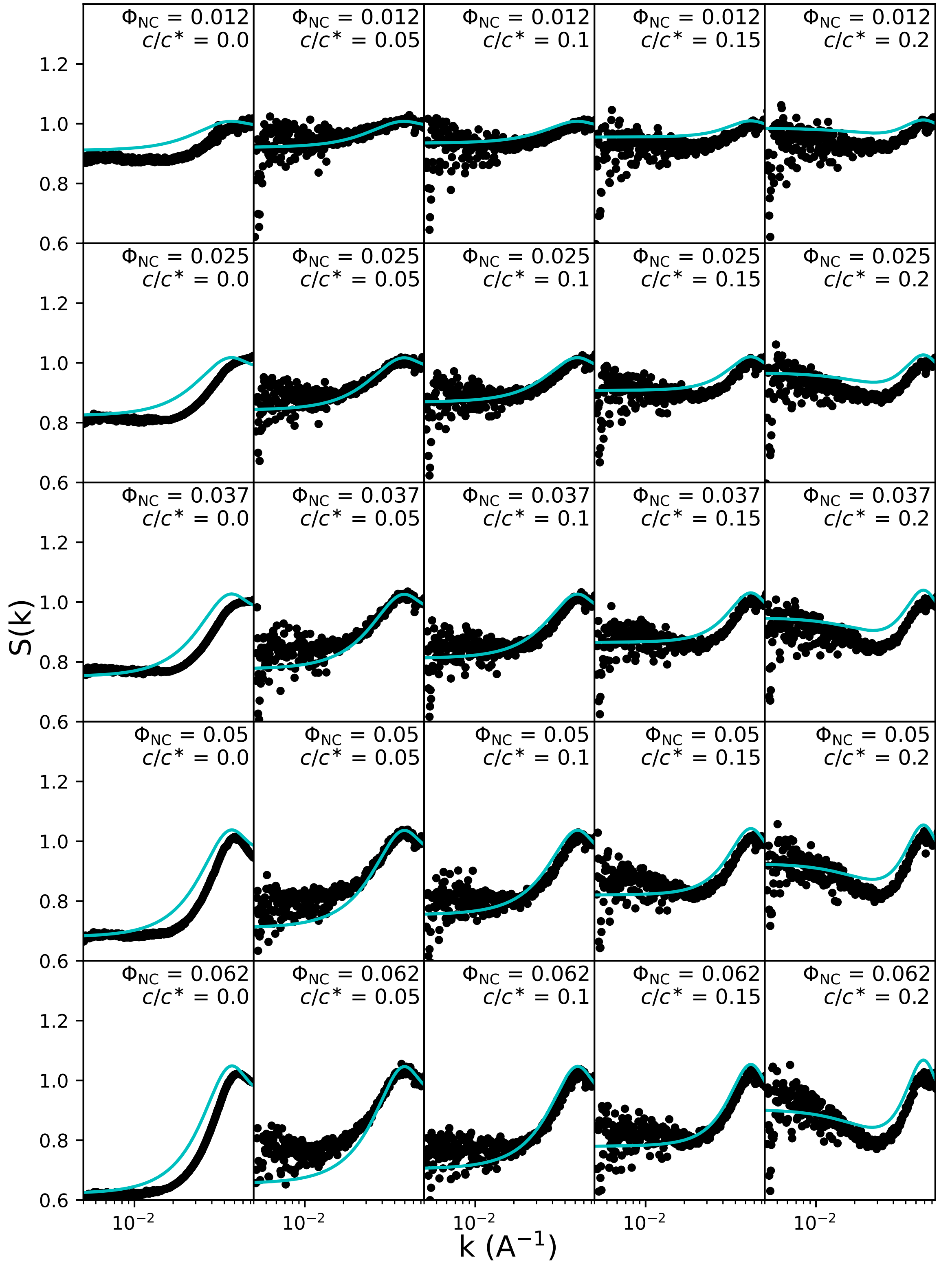

fig. S22. Structure factors from experiment (black dots) and AOV IET (cyan line) at q = 0.17 (σ_{HS} = 10.3 nm, polymer M_w = 1.3 kDa) and the Φ_{NC} and c/c^* indicated.

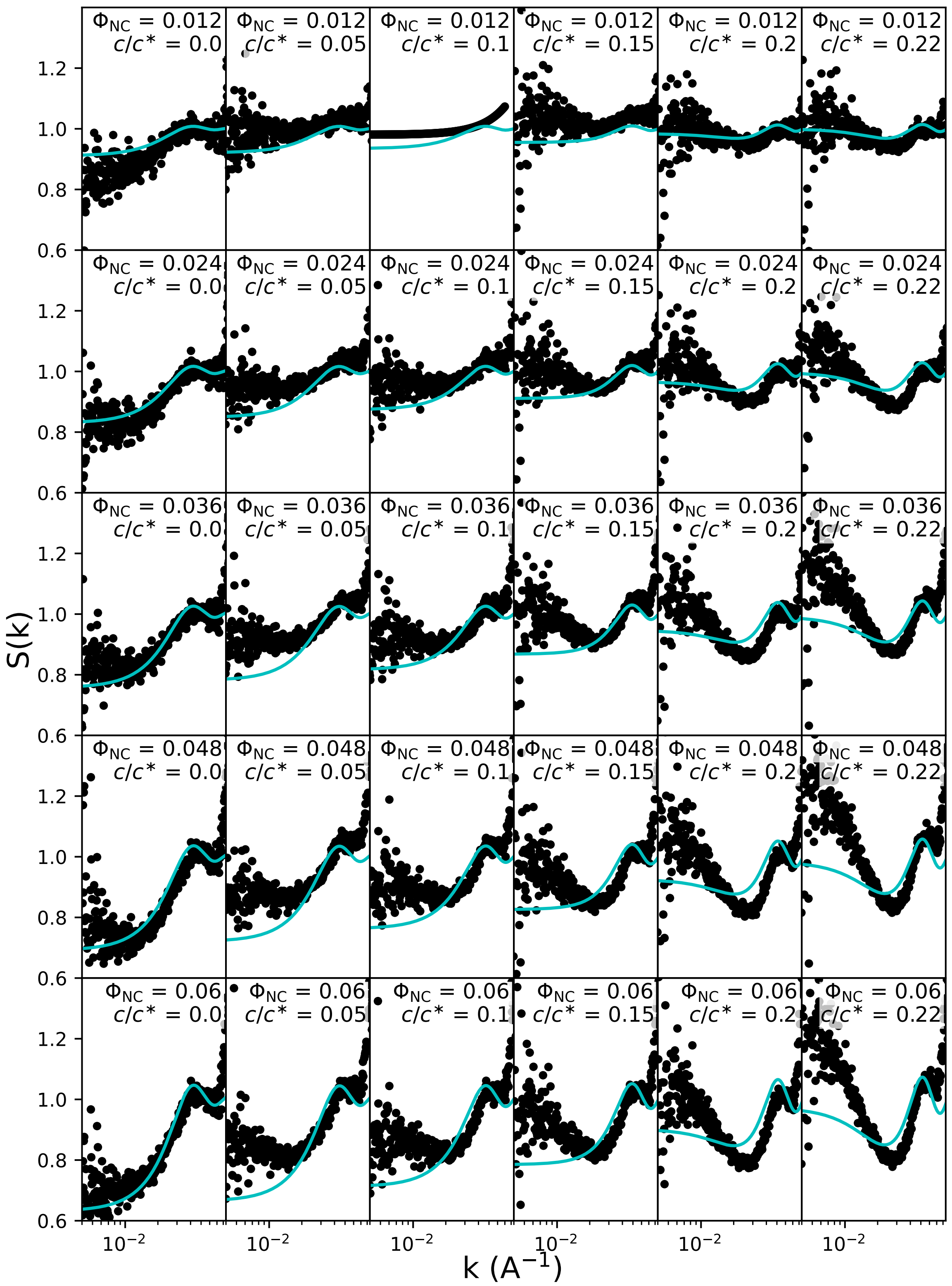

fig. S23. Structure factors from experiment (black dots) and AOV IET (cyan line) at q = 0.17 (σ_{HS} = 13.6 nm, polymer M_w = 2.2 kDa) and the Φ_{NC} and c/c^* indicated.

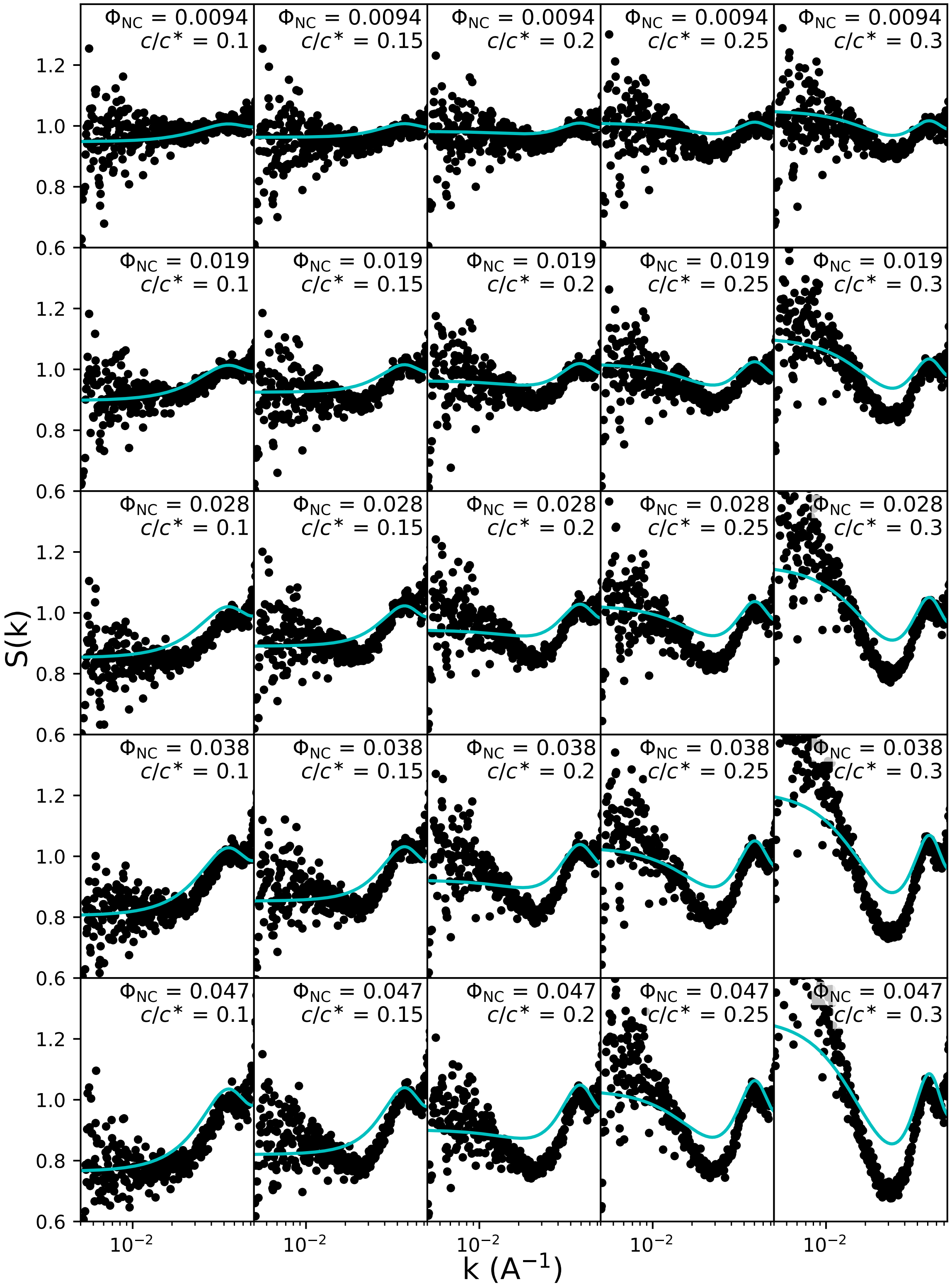

fig. S24. Structure factors from experiment (black dots) and AOV IET (cyan line) at q = 0.2 (σ_{HS} = 11.8 nm, polymer M_w = 2.2 kDa) and the Φ_{NC} and c/c^* indicated.

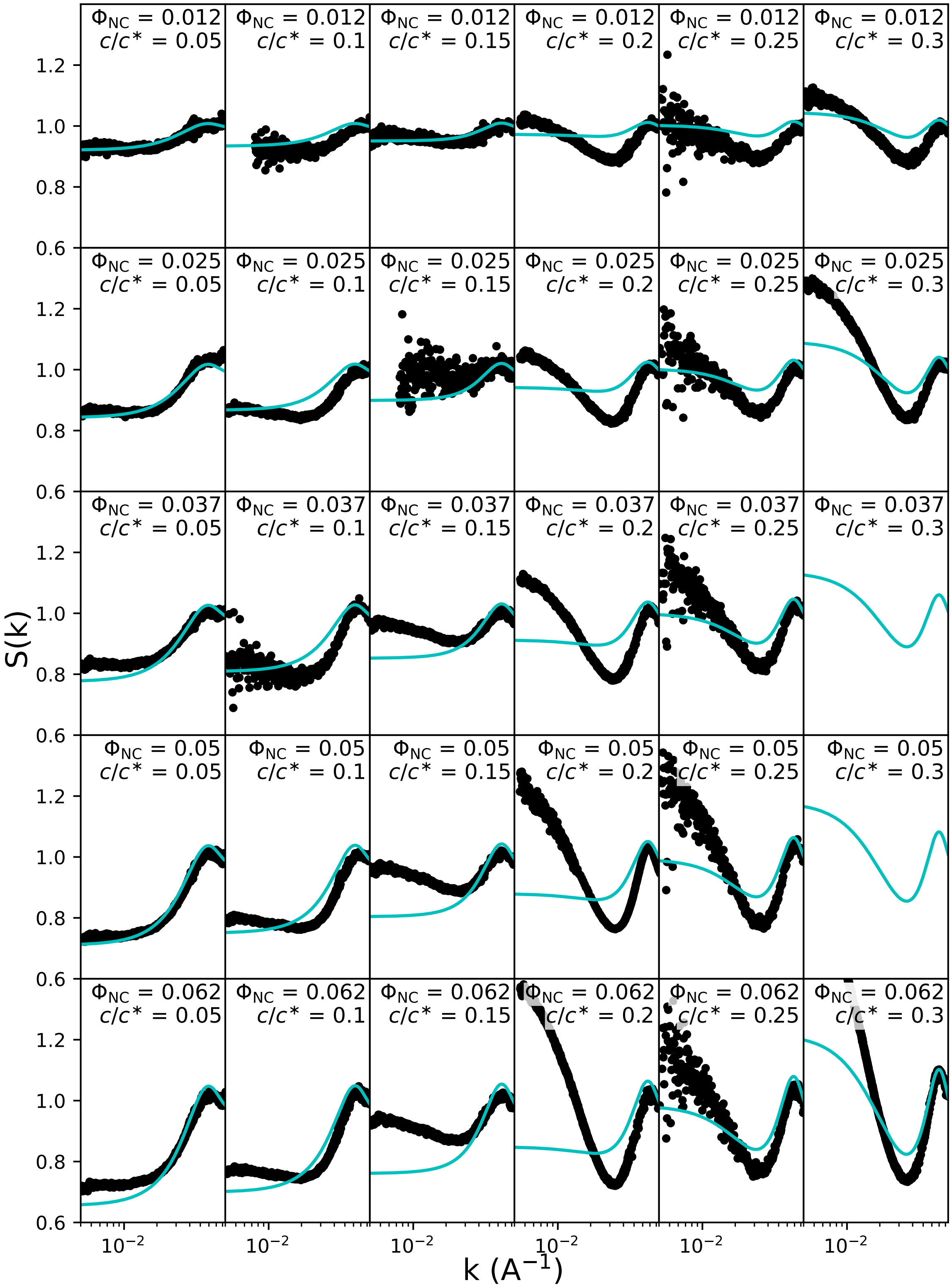

fig. S25. Structure factors from experiment (black dots) and AOV IET (cyan line) at q = 0.23 (σ_{HS} = 10.3 nm, polymer M_w = 2.2 kDa) and the Φ_{NC} and c/c^* indicated.

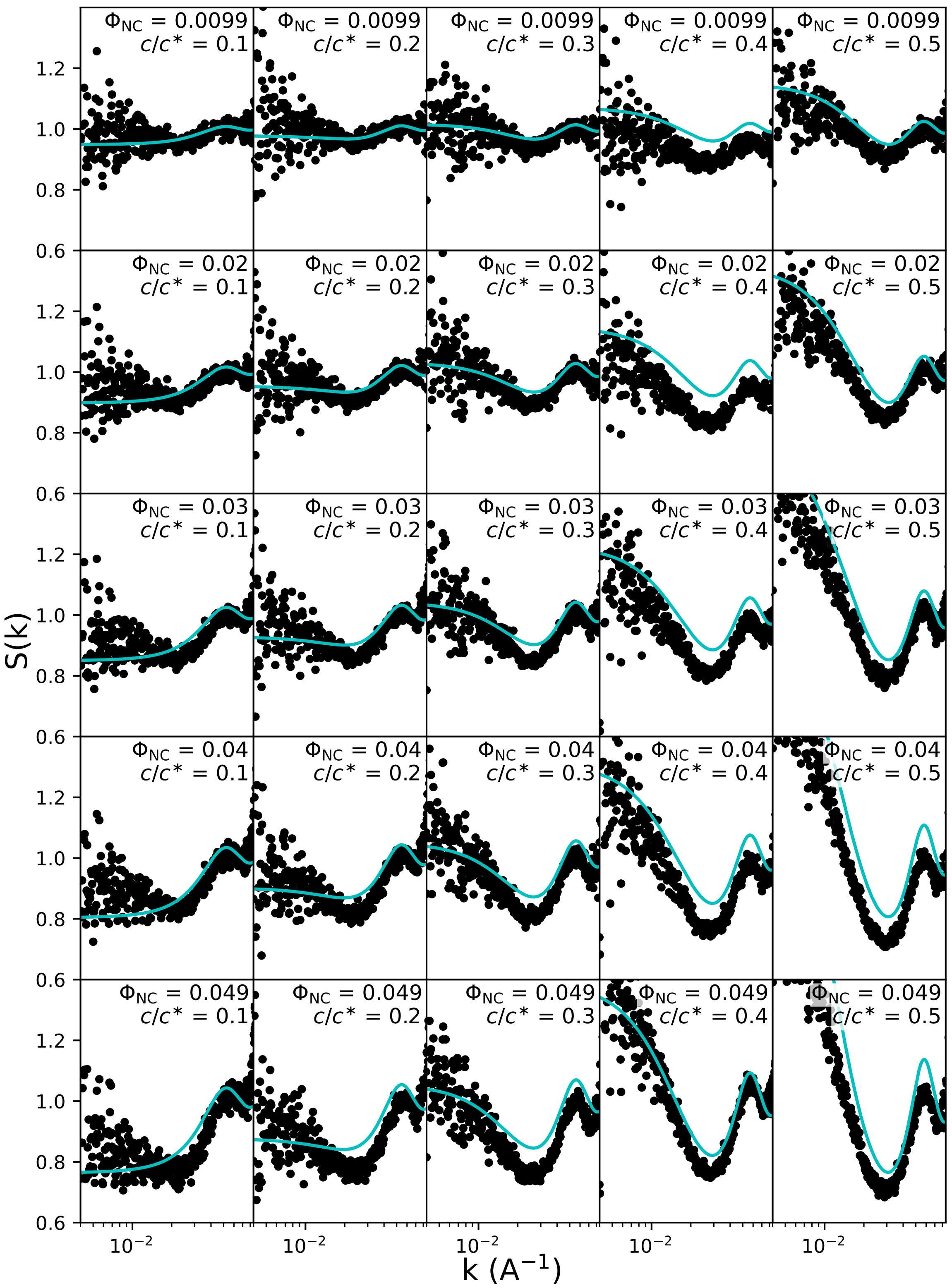

fig. S26. Structure factors from experiment (black dots) and AOV IET (cyan line) at q = 0.55 (σ_{HS} = 11.8 nm, polymer M_{w} = 13 kDa) and the Φ_{NC} and c/c^{*} indicated.

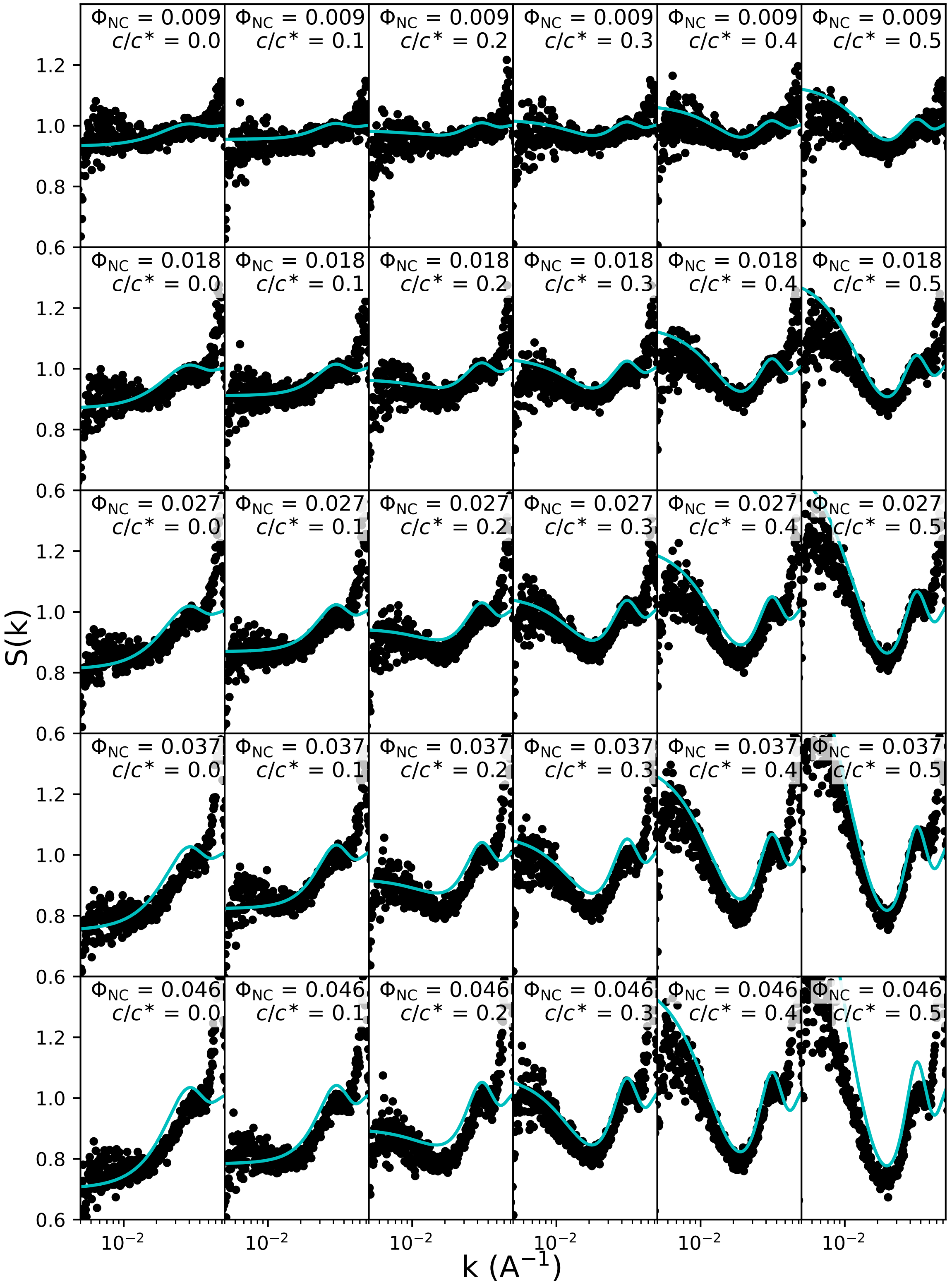

fig. S27. Structure factors from experiment (black dots) and AOV IET (cyan line) at q = 0.66 (σ_{HS} = 14.4 nm, polymer M_w = 25 kDa) and the Φ_{NC} and c/c^* indicated.

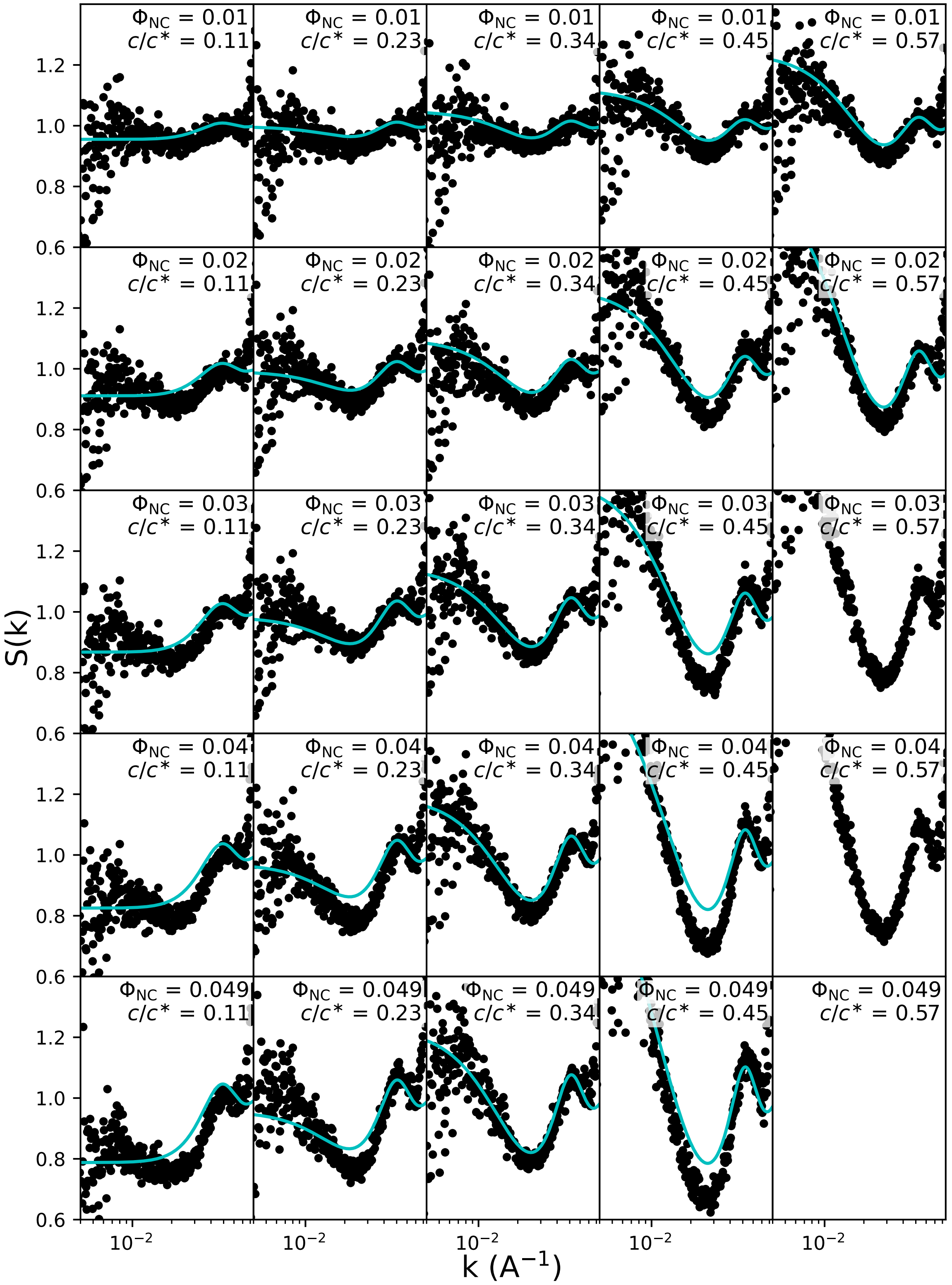

fig. S28. Structure factors from experiment (black dots) and AOV IET (cyan line) at q = 0.76 (σ_{HS} = 12.4 nm, polymer M_w = 25 kDa) and the Φ_{NC} and c/c^* indicated.

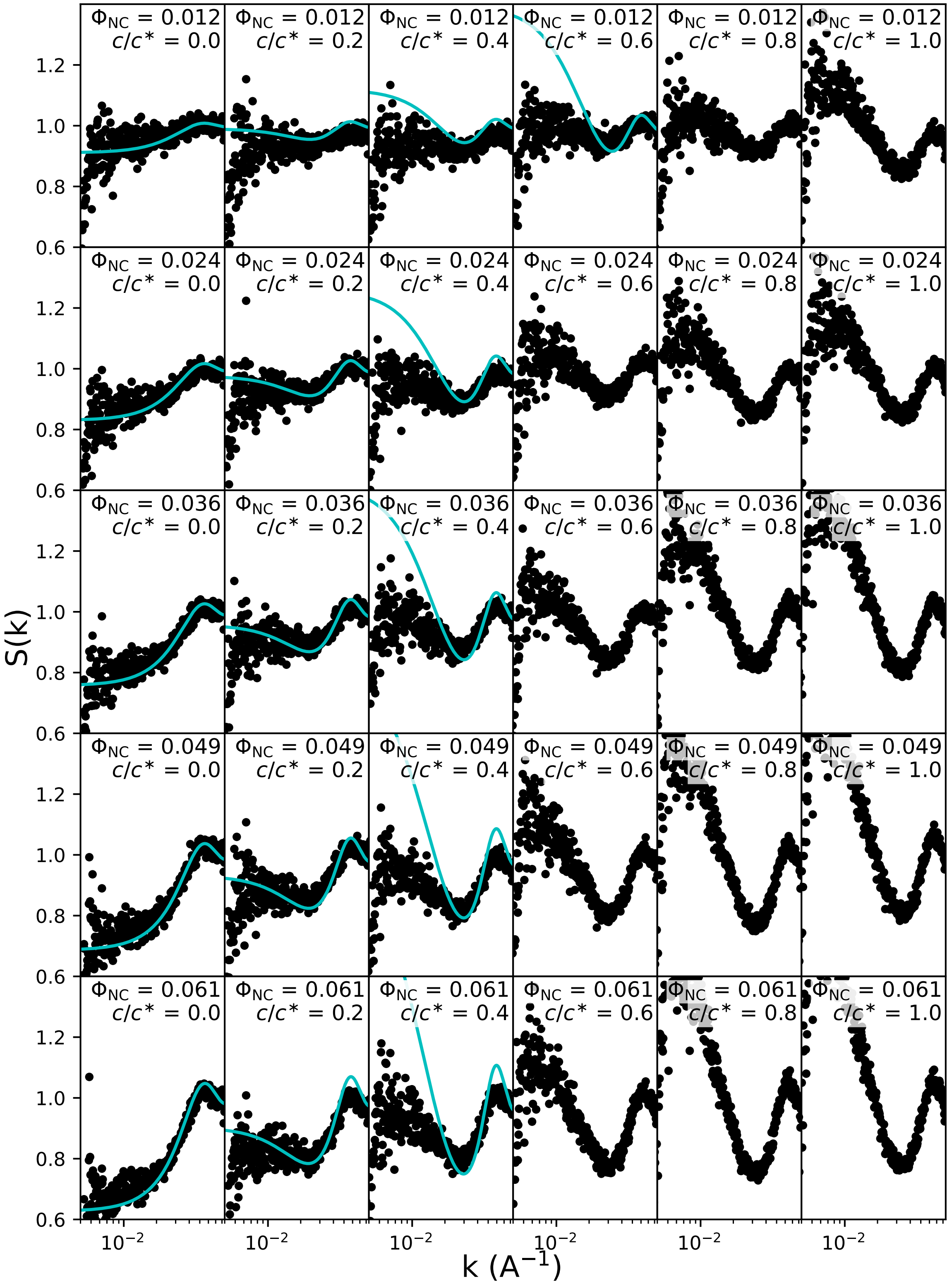

fig. S29. Structure factors from experiment (black dots) and AOV IET (cyan line) at q = 0.89 (σ_{HS} = 10.6 nm, polymer M_w = 25 kDa) and the Φ_{NC} and c/c^* indicated.

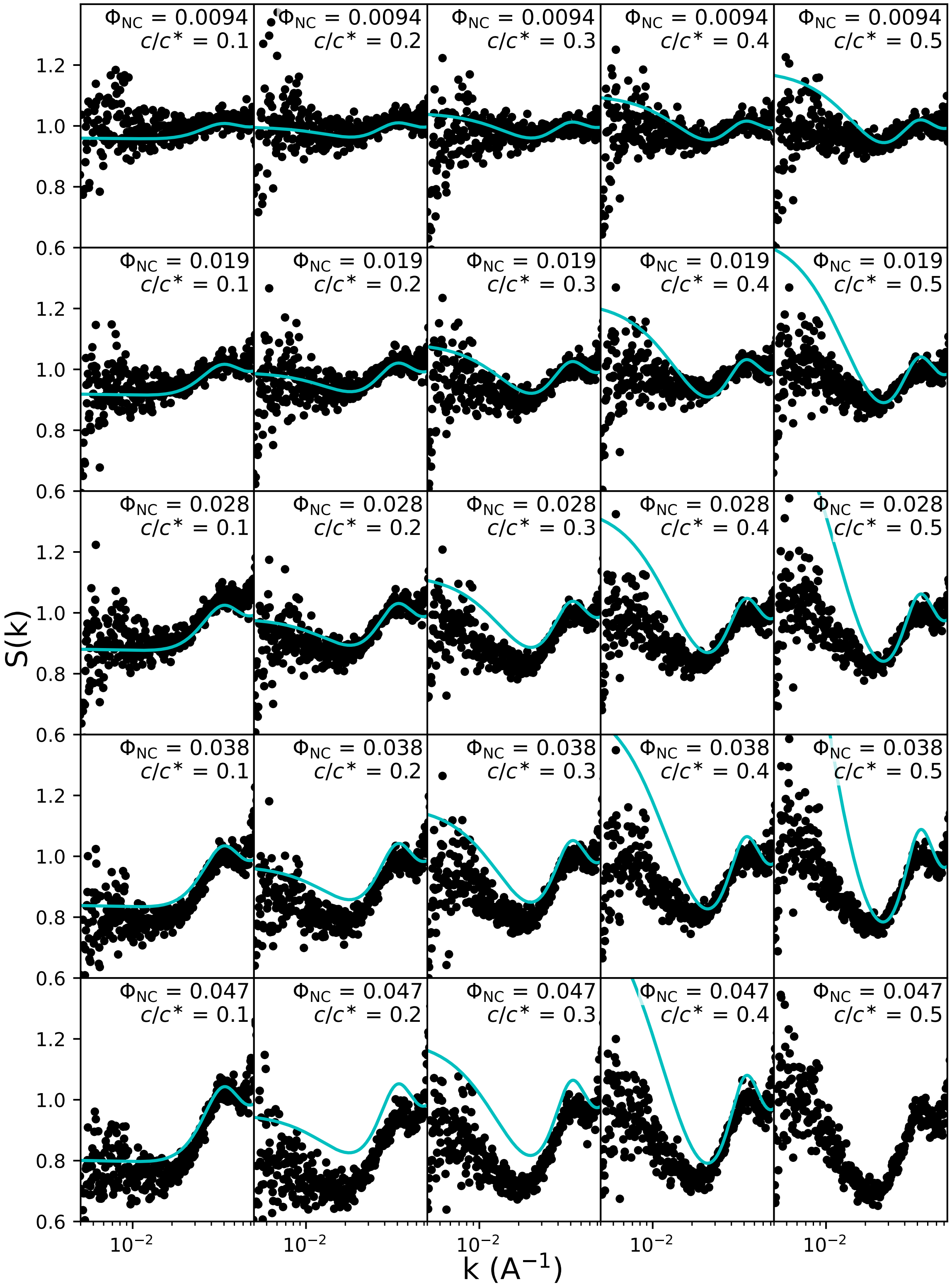

fig. S30. Structure factors from experiment (black dots) and AOV IET (cyan line) at q = 0.98 (σ_{HS} = 11.8 nm, polymer M_w = 35 kDa) and the Φ_{NC} and c/c^* indicated.

7.11 Linear *vs* quadratic fitting for B_2^*

M_{w} (kDa)	q	c/c^*	Linear Fit		R^2	Quadratic Fit	
			B_2^*	B_3^*		B_2^*	B_3^*
1.3	0.17	0.25	-0.35 ± 0.04	—	0.99	-0.48 ± 0.15	0.31 ± 0.33
2.2	0.20	0.30	-1.16 ± 0.09	—	0.99	-0.95 ± 0.22	-0.66 ± 0.69
13	0.55	0.50	-1.26 ± 0.11	—	0.99	-1.62 ± 0.22	0.98 ± 0.59
25	0.67	0.57	-1.83 ± 0.17	—	0.99	-2.37 ± 0.42	1.96 ± 1.47
35	0.89	1.40	-2.30 ± 0.31	—	0.98	-3.24 ± 0.90	3.25 ± 2.94

Table S1. Comparison of linear and quadratic fits for extracting B_2^* across different molecular weights at the highest measured c/c^* before observed phase instability. The linear fits provide a reliable estimation of B_2^*, as indicated by consistently high R^2 values. Quadratic fits introduce an additional fitting parameter, B_3^*, but result in larger uncertainties, suggesting possible overfitting. The third virial coefficient is defined as $B_3^* = B_3/B_3^{HS}$, where $B_3^{HS} = (5/8)\pi^2\sigma_{\mathrm{HS}}^6$ is the hard-sphere reference value. Given the substantial errors associated with B_3^*, we report B_2^* values derived from the linear fit to ensure more robust and interpretable results.
